# Targeting IDH-driven Oncometabolism in glioblastoma: a computational approach for the identification of potential inhibitors

**DOI:** 10.3389/fbinf.2026.1884553

**Published:** 2026-08-26

**Authors:** Nivedhitha Tamilazhagan, Sivakumar Arumugam

**Affiliations:** School of Bio-Sciences and Technology, Vellore Institute of Technology, Vellore, Tamil Nadu, India

**Keywords:** epigenetic modification, glioblastoma, IDH-targeted inhibitors, molecular docking, molecular dynamics simulations

## Abstract

**Background:**

Glioblastoma (GBM) is a malignant brain tumor frequently driven by mutations in isocitrate dehydrogenase (IDH1 and IDH2) enzymes, which promote neomorphic synthesis of the oncometabolite 2-hydroxyglutarate (2-HG) and subsequent metabolic dysfunction. Although these mutations are associated with different clinical outcomes, therapeutic intervention remains challenging due to tumor heterogeneity, cellular plasticity, restricted blood-brain barrier permeability, drug efflux mechanisms, and effective DNA repair pathways. Therefore, this study aims to identify selective inhibitors of mutant IDH proteins by overcoming these challenges.

**Methods:**

10,309 compounds from ChemFaces and MedChemExpress libraries are used for large-scale virtual screening and multi-level ADMET filtering, uncovering 19 lead compounds with favourable drug-likeness. Further, molecular docking followed by 300 ns of molecular dynamics simulations, PCA analysis, and MM-PBSA calculations were performed to explore conformational dynamics.

**Results:**

Molecular docking revealed strong binding affinities of PubChem ID 91457 (I1Hit1) as −7.57 kcal/mol and PubChem ID 4946 (I1Hit2) as −6.82 kcal/mol against IDH1, and PubChem ID 71550939 (I2Hit1) as −11.23 kcal/mol and PubChem ID 4946 (I2Hit2) as −7.87 kcal/mol against IDH2, by outperforming the standard (−6.17 kcal/mol). Molecular dynamics simulations, PCA analysis, and MM-PBSA calculations confirmed complex stability and inhibitory potential. Notably, I1Hit1 and I2Hit1 exhibited enhanced binding free energies of −20.67 ± 2.32 kcal/mol against IDH1 and -28.78 ± 2.67 kcal/mol against IDH2 respectively, compared to the standard, as further supported by computational pharmacophore evaluation.

**Conclusion:**

Collectively, these findings highlight I1Hit1 and I2Hit1 as novel therapeutic compounds with efficient IDH-target inhibition to address epigenetic modification in GBM, and further experimental validation of these compounds is required to demonstrate potential inhibitors of IDH-driven metabolism in GBM.

## 1 Background

Glioblastoma (GBM) is the most aggressive glioma, a glial-origin primary brain tumor, arising predominantly from astrocytes in the cerebral hemispheres. It represents about 45% of all gliomas and nearly 80% of malignant brain tumors (Maity et al., 2025; Kanderi et al., 2025). Gliomas constitute a heterogeneous group of primary central nervous system (CNS) tumors arising from glial cells. Based on cell lineage, they include astrocytomas, oligodendrogliomas, and ependymomas, and are further divided into low and high-grade tumours according to histopathological aggressiveness (Mesfin et al., 2025). The World Health Organisation (WHO) classification system for gliomas is used to grade tumors based on their aggressiveness and growth potential. Low-grade gliomas comprise Grade I and Grade II tumors, which are infiltrative and benign tumors that can undergo progression. High-grade malignant tumors, such as Grade III and Grade IV, specifically Grade IV, including glioblastoma, are highly aggressive, characterised by necrosis and microvascular proliferation, and carry a poor prognosis (Louis et al., 2021; Byun and Park, 2022). GBM is also a WHO Grade IV astrocytoma with tumour proliferation, especially in the temporal and frontal lobes, resulting in sensory and behavioural alterations and contributing to cognitive decline (Balaji et al., 2024). The age-standardised incidence of brain and CNS cancers in India is 2.3 per 100,000 individuals based on WHO estimates (2022) (https://gco.iarc.fr/today/en). GBM has a relatively constant annual incidence in the United States of approximately 3.19 per 100,000 individuals and has a national population with a consistent male predominance (M:F ≈ 1.6:1) (Jovanovich et al., 2024; Prosperetti et al., 2025; Kanderi et al., 2025). It was noted that this disease is rare in children, and the pediatric incidence remains low (∼0.85 per 100,000) (Grochans et al., 2022). However, GBM is associated with poor prognosis, with reported median survival ranging from 10 to 15 months in Indian cohorts (Mohammed et al., 2022; Tali et al., 2023).

Recent studies have identified mutations in the Isocitrate dehydrogenase (IDH) genes in various solid tumours, including glioma, cholangiocarcinoma, and chondrosarcoma (Carosi et al., 2024). IDH enzymes are key players of cellular metabolism, catalysing oxidative decarboxylation of isocitrate to α-ketoglutarate (α-KG) within the Krebs cycle, thus modulating redox balance and supporting biosynthetic processes. Among the three isoforms of IDH1, IDH2, and IDH3, mutations in IDH1 and IDH2 are of oncogenic relevance. These somatic gain-of-function mutations result in the aberrant production of the oncometabolite 2-hydroxyglutarate (2-HG), which disrupts cellular differentiation and epigenetic regulation (Balaji et al., 2024; Carosi et al., 2024). GBM is molecularly stratified into IDH-wildtype and IDH-mutant subtypes, with IDH1 mutations predominantly at the R132 residue, comprising variants such as R132H, R132C, R132S, R132G, and R132L, which are frequently observed in secondary GBM with an incidence rate of >70% (Murugan and Alzahrani, 2022). This most prevalent IDH1 R132H mutation accounts for approximately 89% of cases, while other mutations occur less frequently (Bajaj et al., 2025). IDH2 mutations typically occur at R140 and R172 residues, including substitutions like R140Q, R172G, R172K, R172M, R172S, and R172T (Carosi et al., 2024). Co-occurring genetic alterations in GBM include TP53, ATRX, and TERT promoter mutations, as well as aberrations in BRAF, FGFR1, EGFR, MGMT, and WT1. The mutant enzymes remain a pathogenic driver of oncogenic signalling by causing the accumulation of 2-HG, which disrupts α-KG-dependent dioxygenase, leading to epigenetic dysregulation and metabolic reprogramming that sustain tumour growth. Therapeutic inhibition of mutant IDH enzymes aims to suppress 2-HG driven tumour promoting pathways and restore cellular regulatory balance. Targeting mutant IDH enzymes is therefore a rational and promising strategy to modulate downstream metabolic and epigenetic effects (Carosi et al., 2024).

The blood-brain barrier (BBB) is a selectively permeable protective system that regulates molecular transport into the CNS. The BBB significantly restricts the delivery of most chemotherapeutic drugs to brain tumors, including glioblastoma, despite being crucial for preserving neuronal homeostasis and therefore by limiting the passage of large and hydrophilic molecules (Teraiya et al., 2023; Porosk and Langel, 2025). Despite the challenges in treating GBM, certain drugs that can penetrate the BBB, such as Temozolomide, Lomustine, Irinotecan, Vorasidenib, ivosidenib and Cisplatin, are utilized in GBM chemotherapy. Moreover, small-molecule inhibitors targeting mutant isocitrate dehydrogenase enzymes have emerged as a promising approach for addressing mutant IDH cancers. Vorasidenib and ivosidenib act as inhibitors of mutant IDH enzymes and have demonstrated initial clinical effectiveness in treating mutant IDH glioma (Mellinghoff et al., 2023). Ivosidenib, an inhibitor targeting mutant IDH1, has received approval for the treatment of certain mutant IDH1-positive acute myeloid leukemia cases, as well as for patients with previously treated, locally advanced or metastatic cholangiocarcinoma. Additionally, it has exhibited initial antitumor effects in patients with mutant IDH1 glioma and chondrosarcoma (Tap et al., 2020). Vorasidenib, a dual inhibitor of mutant IDH1 and IDH2 enzymes, was specifically developed to enhance BBB penetrance and has demonstrated preliminary antitumor activity in patients with mutant IDH glioma. In a peri-operative study, Vorasidenib demonstrated a reduction of over 90% in 2-HG levels within resected tumour tissue, accompanied by reversal of gene expression patterns and epigenetic modifications commonly linked to IDH-mutant gliomas (Mellinghoff et al., 2023). In parallel with small-molecule IDH inhibition, gene therapy approaches are emerging as an alternative translational strategy for glioma, including AAV-mediated dual-payload vector platforms currently entering early-phase clinical evaluation for high-grade glioma (NCT07346144), illustrating the evolving shift toward multimodal therapeutic interventions capable of addressing the genetic and microenvironmental complexity of GBM, which may require therapeutic strategies extending beyond IDH-targeted monotherapy. Despite significant advances in therapeutic development, GBM continues to be associated with a high mortality rate, with a median survival of approximately 14 months following standard treatment comprising surgical resection, radiotherapy, and temozolomide chemotherapy, and a 5-year survival rate below 5% (Balaji et al., 2024; Angom et al., 2023). The limited efficacy of current treatment approaches is attributed to multiple interrelated factors, including tumour heterogeneity, cellular plasticity, BBB impermeability, drug efflux pump activity, glioma stem cell resilience, and DNA damage repair mechanisms, all of which collectively impair therapeutic drug delivery and target engagement at the tumour site. These poor clinical outcomes highlight the need for more effective therapeutic strategies. More advanced computational modelling and integrative analytical methodologies hold promise for the highly invasive and complex molecular nature of GBM, aiding the development of targeted and individualised therapeutic strategies (Fedorova et al., 2023).

**FIGURE 1:**
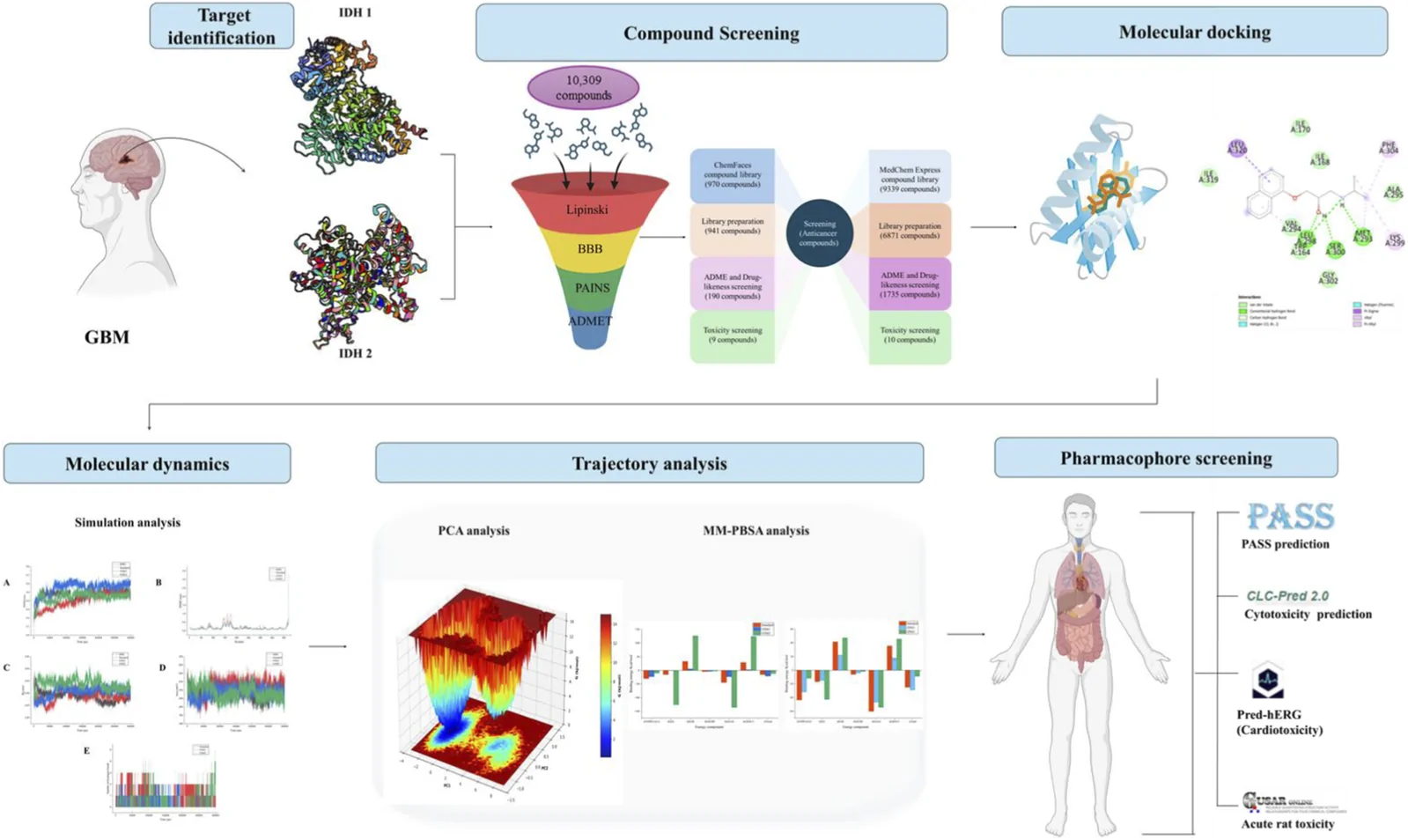
An overview of study design illustrating computational analyses involving compound screening, molecular docking, molecular dynamics simulations and post-simulation analysis. This figure was created using https://www.biorender.com/.

Building on recent advances in IDH-targeted therapies, the present study aims to identify and characterise potential inhibitors capable of limiting GBM progression with improved safety profiles. To address this objective, an integrated *in silico* framework was employed to virtually screen compounds retrieved from chemical libraries, prioritising drug-like molecules based on predicted ADMET and pharmacokinetic properties. Top-ranked compounds were subsequently evaluated for their binding affinity and interaction profiles with mutant IDHs using molecular docking, followed by molecular dynamics simulations, principal component analysis, and MM-PBSA calculations to assess conformational stability and binding efficacy. Furthermore, comprehensive *in silico* toxicity assessments, including hepatotoxicity, cardiotoxicity, cytotoxicity, and acute toxicity modelling were performed to identify compounds with reduced adverse-effect potential as represented in Figure 1. Collectively, this study focuses on the discovery of promising IDH inhibitors that may offer effective control of GBM tumour progression with enhanced safety and improved tolerability.

## 2 Materials and methods

### 2.1 Protein retrieval and preparation

The protein structures are retrieved from the Protein Data Bank (https://www.rcsb.org/) of mutant IDH 1 or Isocitrate dehydrogenase [NADP] cytoplasmic (PDB:6VEI) or IDH1 and mutant IDH 2 or Isocitrate dehydrogenase [NADP] mitochondrial (PDB: 6VFZ) or IDH2 based on the literature (Balaji et al., 2024) and were subjected to docking with screened compounds. The X-ray crystallographic structure of the IDH1 and IDH2 target proteins was retrieved from Protein Data Bank (https://www.rcsb.org/) with a resolution of 2.10 Å, R-free value of 0.195, R-work value of 0.158, and resolution of 1.99 Å, R-free value of 0.209, R-work value of 0.177, respectively. Protein energy minimisation was performed using the steepest descent method for 100 steps with a step size of 0.02 Å. Furthermore, this protein structure was refined by eliminating water molecules, co-crystallised ligands, and heteroatoms using BIOVIA Discovery Studio. The processed IDH1 and IDH2 proteins were then saved in PDB format for subsequent molecular docking studies.

### 2.2 Ligand preparation

A collection of 10,309 compounds was downloaded in CSV format from two open-source chemical libraries of anti-cancer compounds, namely ChemFaces (https://chemfaces.com/screening/index.php) and MedChem (https://www.medchemexpress.com/screening-libraries.html). These compounds were then passed through preliminary screening, followed by ADMET screening. The SDF files of the screened compounds and 3D conformers were obtained and downloaded from PubChem (https://pubchem.ncbi.nlm.nih.gov). Subsequently, these SDF files were subjected to energy minimisation by means of Avogadro 1.2.0 software on the MMFF94 force field using 100 steps and saved as PDB files. Then the energy-minimised PDB files were converted to a PDBQT file by using Open Babel for docking.

### 2.3 ADMET screening

One of the primary reasons for the failure of drug discovery endeavours is associated with suboptimal pharmacokinetics and toxicity concerns. Consequently, it is essential to assess the Absorption, Distribution, Metabolism, Excretion, and Toxicity (ADMET) characteristics of compounds during the early stages of drug development. In this study, ADMET lab 2.0 and Swiss ADME software tools were employed to predict the ADMET profiles of IDH inhibitors (Zhao et al., 2024). The platform automatically generated predictions related to ADMET properties by providing the SMILES strings of the compounds. Furthermore, the ADMET lab 2.0 and Swiss ADME servers facilitated a comprehensive analysis of pharmacokinetic parameters to evaluate the detailed ADMET characteristics (Das et al., 2024).

### 2.4 Molecular docking

The molecular docking analysis of the protein-ligand complex was performed by using AutoDockTools-1.5.7 version. Polar hydrogens and Kollman charges were incorporated into the protein targets. The protein-ligand complex was subjected to site specific docking with active site based on literature (Balaji et al., 2024) for the protein 6VEI (TYR272, VAL276, ASP273, ILE251, VAL255, GLN277, TRP267, SER280, TRP124, ALA258, VAL281, and MET259) and for the protein 6VFZ (VAL315, TYR311, ASP312, ILE290, GLN316, ILE319, TRP306, VAL294, VAL297, LEU298, LEU320, and TRP164) while generating a grid map using AutoDockTools-1.5.7. The dimensions of the grid box for IDH1 (PDB ID:6VEI) were configured as X = 124 Å, Y = 80 Å, and Z = 94 Å, with spatial co-ordinates of 49.595, 20.92, and 24.604, respectively. Conversely, the grid box parameters for IDH2 (PDB ID: 6VFZ) were established as X = 104 Å, Y = 106 Å, and Z = 110 Å, with spatial centre co-ordinates positioned at −24.563, 10.398, and −9.616, respectively. The grid parameter file (GPF) was generated to define parameters for Autogrid to precompute affinity maps for molecular docking. The Lamarckian genetic algorithm (LGA) is the primary search algorithm used in Autodock for predicting ligand-receptor binding conformations. The docking parameter file (DPF) was configured to use the Lamarckian genetic algorithm for certain runs and population sizes. The docking log file (DLG) was generated to analyse docking poses, binding stability, and interaction patterns. The search algorithm systematically scanned through a series of ligand geometries and interactions by eliminating some configurations based on their relative binding energy. Binding preferences are determined as binding affinities based on the Gibbs free energy (ΔG) calculated by Autodock algorithms, where a more negative ΔG value indicates stronger affinity (Vural and Kaymak, 2025). The lowest docking conformer was selected at the pose having minimal predicted binding free energy (ΔG), and thus, being most favoured for binding. To assess the interactions between ligands and proteins, the final docking poses were analysed with PyMOL and BIOVIA Discovery Studio Visualizer. The evaluation concentrated on the basic molecular forces in the active site (hydrogen bonding, hydrophobic contacts, electrostatic interactions and van der Waals forces) for finding better compounds with favourable orientation and binding energy (Rashwan et al., 2025).

### 2.5 Molecular dynamics simulation

Molecular dynamics (MD) simulation is required to determine the protein flexibility and to validate the binding stability between the protein-ligand complex. According to the result obtained from molecular docking analysis, the IDH1, IDH2, standard and hit compounds of protein-ligand complexes are selected for evaluation under dynamic conditions (Poustforoosh, 2025). In this study, the GROMACS 2023.1 version was used for exhaustive molecular dynamics simulation at 300 ns (Zhao et al., 2024). The SwissParam online web server (https://old.swissparam.ch/) was used to generate a ligand topology file by uploading a ligand mol2 file compatible with the CHARMM force field (Subramani and Venugopal, 2024). The CHARMM 27 force field was chosen with the TIP3P solvent model, which was implemented in an orthorhombic box at constant temperature and pressure conditions, with periodic boundary conditions to maintain system stability (Zhao et al., 2024). The protein was positioned at the centre of a cubic simulation box with a minimum distance of 1 nm from the box boundary (Pradeep et al., 2025). Sodium (Na^+^) and chloride (Cl^−^) ions were added to neutralise the system and maintain electrostatic stability (Bhrdwaj et al., 2023; Swati et al., 2025). The energy minimization was carried out to relax steric overlaps by using the steepest descent algorithm for 50,000 steps. The system was equilibrated under NVT conditions at 300K, followed by NPT equilibration at 300K and 1 bar to stabilize temperature and pressure before production. Temperature coupling was achieved using a Berendsen thermostat, while pressure coupling was controlled by Parrinello-Rahman barostat. The final production MD simulation was performed for 300 ns with a 2 fs (0.002 ps) time step (Sharma and Arumugam, 2025). The post-simulation analysis was performed using GROMACS by calculating various parameters such as Root-mean-square deviation (RMSD) to measure the overall structural stability and equilibration (Ormeño and General, 2024), Root-mean-square fluctuation (RMSF) to estimate the flexibility of residues or atoms based on their positional displacements over the course of a simulation in a complex molecule, Radius of Gyration (Rg) to evaluate molecular compactness and reflects folding/unfolding behaviour and conformational changes, Solvent Accessible Surface Area (SASA) quantifies the extent of protein surface exposed to solvent, providing insight into structural stability and binding interactions and Hydrogen bond (H-bond) analysis examine hydrogen bond formation in protein-ligand complex (Hollingsworth and Dror, 2018). These parameters were analyzed using GROMACS tool (gmx rms, gmx rmsf, gmx gyrate, gmx sasa, and gmx hbond) and the resulting plots were visualized using XMgrace and Origin pro server.

### 2.6 Principal component analysis (PCA)

PCA is a multivariate statistical analysis used to reduce the dimensionality of complex datasets by extracting the principal modes of variance. PCA enables the identification of the predominant collective motions of protein-ligand complexes, facilitating the analysis of conformational dynamics and structural stability (Post et al., 2019; Thenier-Villa et al., 2024). For PCA, the simulation trajectories were aligned to a reference structure by least-squares fitting, thereby minimizing overall motion and emphasizing internal structural fluctuations. The covariance matrix of C-α atomic positional fluctuations are calculated from the fitted trajectory using the “covar” module in GROMACS, and diagonalized to give eigenvalues and eigenvectors (David and Jacobs, 2014). The “anaeig” tool were used subsequently to analyze collective motions by projecting the trajectory onto principal components with individual eigenvectors selected for 2D plotting projections. The “gmx sham” command was employed to examine the essential movements of the core atoms of proteins and elucidate their impact on ligand interaction by constructing the Free Energy Landscape (FEL) (Dubey et al., 2025a). The FEL plot was used to examine the structural stability and conformational dynamics of biomolecular systems (Dubey et al., 2025b). The 3D FEL plot was produced to reveal the distribution of conformational states, with distinct narrow and wide energy basins at different energy levels (Dubey et al., 2025a).

### 2.7 MM-PBSA analysis

Molecular Mechanics Poisson-Boltzmann Surface Area (MM-PBSA) is a computational approach that combines molecular mechanics energies with continuum solvent models to estimate binding free energy. In our study, the gmx_MMPBSA tool was employed to quantify and analyse the individual energetic contributions associated with molecular binding and stability (Tharamelveliyil Rajendran et al., 2024). The last 20 ns (2000 frames) of the MD simulation trajectory of standard and hit compounds were selected for MMPBSA analysis to evaluate the complex stability during the simulation. The total binding free energy was calculated from individual energy components using the following equation:

$$\Delta G_{\text{binding}}=\Delta G_{\text{Gas}}\;\left(\Delta E_{\text{EL}}+\Delta E_{\text{VDWAALS}}\;\right)+\Delta G_{\text{solv}}\;\left(\Delta E_{\text{GB}}+\Delta E_{\text{SURF}}\;\right)$$

Where gas-phase energy (G_Gas_) comprises the combined contribution of electrostatic energy (EEL) and van der Waals (VDWAALS) interactions, while solvation-free energy (G_solv_) includes polar solvation energy (EGB) and non-polar solvation energy (ESURF) (Sharma et al., 2025).

These components were used to estimate the total binding free energy using the following equation:

$$\Delta G_{\text{binding}}=G_{\text{complex}}-\left(G_{\text{protein}}+G_{\text{ligand}}\;\right)$$

Where G_complex_ represent the binding free energy of protein-ligand complex, G_protein_ and G_ligand_ denote the free energies of protein and ligand in the unbounded state, respectively. The significance of the observed values was determined through statistical analysis and reported as mean and standard deviation. Furthermore, the gmx_MMPBSA_ana module was employed to facilitate the graphical representation and interpretation of the results obtained from the gmx_MMPBSA analysis.

### 2.8 Toxicity prediction

The toxicity prediction was performed for the identified hit compounds, as toxicity arising from interactions with multiple biological targets remains a major cause of drug candidate attrition during development (Poroikov et al., 2007; Cavasotto and Scardino, 2022). The PASS (Prediction of Activity Spectra for Substances) web tool (https://www.way2drug.com/passonline/predict.php) was employed to evaluate possible biological activities; predictions of the probability of active (Pa) exceeding the probability of inactive (Pi) were considered as biologically significant (Alghamdi et al., 2025; Vural and Kaymak, 2025). Additionally, *in silico* cytotoxicity was evaluated using CLC-Pred 2.0 (https://www.way2drug.com/CLC-pred/index.php), which applies structure-activity relationship analysis to estimate cytotoxic potential across cell lines (Lagunin et al., 2023). Cardiotoxicity majorly affects the drug attrition during pharmaceutical development (Passini et al., 2017). Many inhibitors induce cardiac arrhythmias by blocking hERG potassium channel, which plays a critical role in cardiac repolarization. Notably, hERG inhibition is associated with long QT syndrome (LQTS) and an increased risk of sudden cardiac death (Delre et al., 2022). Therefore, early identification of hERG blockers helps to prevent late-stage drug failure and ensure safety during drug development (Li et al., 2025). Cardiotoxicity prediction was examined using *in silico* tools like Pred-hERG 5.0 (https://predherg.labmol.com.br/), which helps to predict the probability of a compound blocking the hERG channel using binary classification, multiclass prediction, probability score, and map (Sanches et al., 2024). *In vivo* toxicity prediction using the GUSAR online tool (https://www.way2drug.com/gusar/acutoxpredict.html) is essential for early, reliable, and ethical assessment of lead compound safety. The server provides LD50 values for multiple administration routes, toxicity class, applicability domain (AD) information, and model statistics (Peter et al., 2025).

## 3 Results

### 3.1 ADMET analysis

The 10,309 compounds were retrieved from ChemFaces database (970 compounds) and the MedChem Express database (9339) of anti-cancer compounds used for computational structure-based investigations. In 970 compounds from the Chemfaces screening library, 941 compounds were subjected to ADME screening, where 585 compounds passed the Lipinski rule of five, 275 compounds passed BBB, 209 compounds passed PAINS, 192 compounds passed bioavailability, 190 compounds passed GI absorption, 132 compounds passed mutagenicity, 63 compounds passed carcinogenicity, 21 compounds passed toxicophore, 17 compounds passed H-HT, 9 compounds passed DILI. In 9339 compounds from the Medchem Express compound screening library, 6871 compounds were refined based on invalid smiles and subjected to ADMET screening. 1735 compounds were screened based on the Lipinski rule of five, BBB, PAINS, bioavailability, GI absorption, and TPSA. Further, among 1735 compounds, 10 compounds are screened based on mutagenicity, carcinogenicity, toxicophore, H-HT, and DILI. According to ADMET 2.0, the probability values from 0 to 0.3 are represented as non-mutagenic, non-carcinogenic, non-hepatotoxic, and negative to DILI, which are depicted in Table 1. Conclusively, a total of 19 compounds from both libraries were subjected to docking studies as screened compounds. The ADME screening, comprehensive molecular properties, and drug-likeness evaluation for hit compounds are represented in Tables 2,3. Supplementary Tables S1–S3 present the results of ADME screening, detailed molecular characteristics, drug-likeness assessment, and toxicity evaluations for the screened compounds. Furthermore, to evaluate the BBB permeability of the identified hit compounds, BOILED-egg analysis was performed using SwissADME, predicting the relationship between lipophilicity (WLOGP) and topological polar surface area (TPSA) to predict passive BBB and gastrointestinal permeation, with P-glycoprotein (P-gp) substrate indicated by point colour depicted in Supplementary Figure S1. The standard compound, vorasidenib was positioned within the BBB-permeant yolk region and predicted as a non-substrate of P-gp (PGP-). Both I1Hit1 and I2Hit1 were similarly positioned with the yolk region and predicted as PGP-, consistent with their favourable TPSA and LogP values reported. I1Hit2&I2Hit2 were also positioned with BBB-permeant yolk region and predicted as PGP-, this consistent PGP- classification suggests that these predicted compounds avoid active efflux at BBB, supporting their theoretical suitability for CNS targeted delivery in glioblastoma.

**TABLE 1:** Predicted Toxicity profile for screened hit compounds.

S.no	Compound & PubChem ID	Mutagenicity	Carcinogenicity	Toxicophore	H-HT	DILI
1	Vorasidenib Standard (117817422)	0.009 (Inactive)	0.199 (Inactive)	0	0.99 (Active)	0.916 (Active)
2	β-Eudesmol I1Hit1 (91457)	0.009 (Inactive)	0.069 (Inactive)	0	0.047 (Inactive)	0.087 (Inactive)
3	Isocoronarin D I2Hit1 (71550939)	0.123 (Inactive)	0.292 (Inactive)	0	0.023 (Inactive)	0.166 (Inactive)
4	Propranolol I1Hit2 & I2Hit2 (4946)	0.064 (Inactive)	0.155 (Inactive)	0	0.115 (Inactive)	0.017 (Inactive)

**TABLE 2:** ADME-based screening of hit compounds.

S.no	Compound& PubChem ID &	Lipinski rule of five	BBB permeant	PAINS	Bioavailability score	GI absorption
1	Vorasidenib Standard (117817422)	Yes	Yes	0	0.55	High
2	β-Eudesmol I1Hit1 (91457)	Yes	Yes	0	0.55	High
3	Isocoronarin D I2Hit1 (71550939)	Yes	Yes	0	0.55	High
4	Propranolol I1Hit2 & I2Hit2 (4946)	Yes	Yes	0	0.55	High

**TABLE 3:** Comprehensive molecular properties and Drug-Likeness evaluation for screened hit compounds.

S.no	Compound & PubChem ID	Molecular weight	HBD	HBA	Log p	TPSA	Rotatable bond count	Ghose	Veber	Egan	Muegge
1	Vorasidenib Standard (117817422)	414.74	2	10	5.29	75.62	7	1	0	1	1
2	β-Eudesmol I1Hit1 (91457)	222.37	1	1	3.74	20.23	1	0	0	0	1
3	Isocoronarin D I2Hit1 (71550939)	318.45	1	3	4.36	46.53	2	0	0	0	0
4	Propranolol I1Hit2 & I2Hit2 (4946)	259.34	2	3	2.98	41.49	6	0	0	0	0

### 3.2 Molecular docking

**FIGURE 2:**
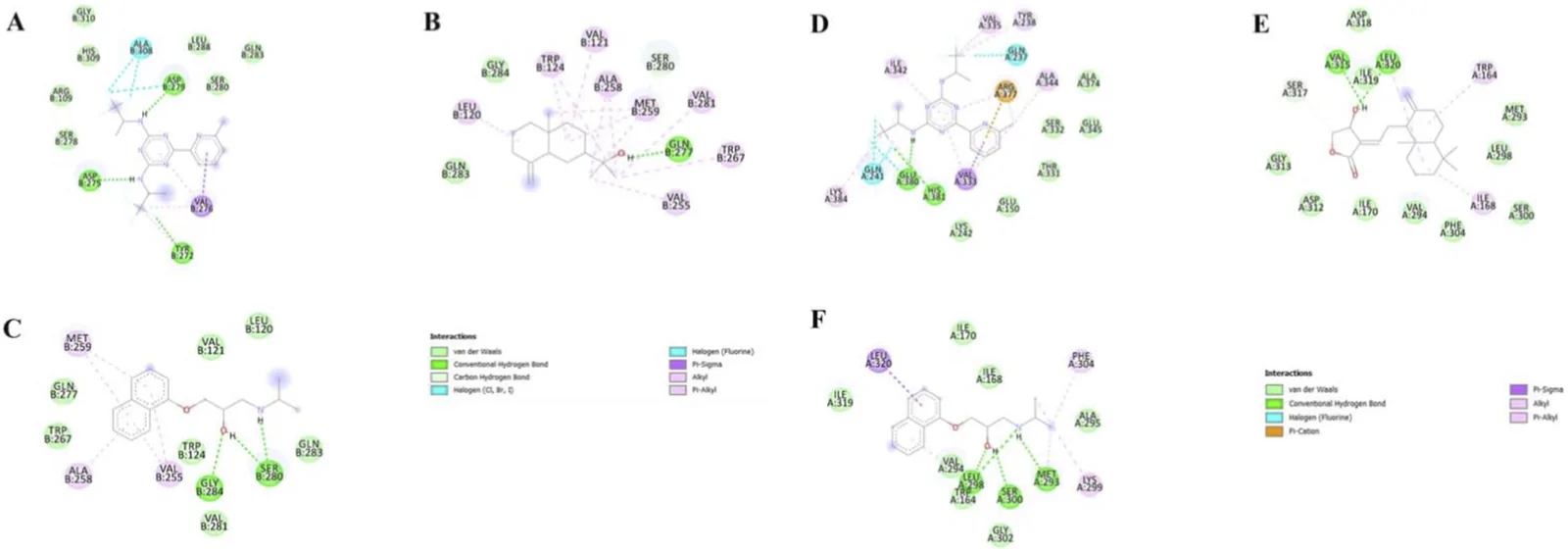
2-D visualization of protein-ligand interaction of IDH1 (left panel) **(A)** Standard, **(B)** I1Hit1 and **(C)** I1Hit2 and IDH2 (right panel) **(D)** Standard, **(E)** I2Hit1, and **(F)** I2Hit2.

**FIGURE 3:**
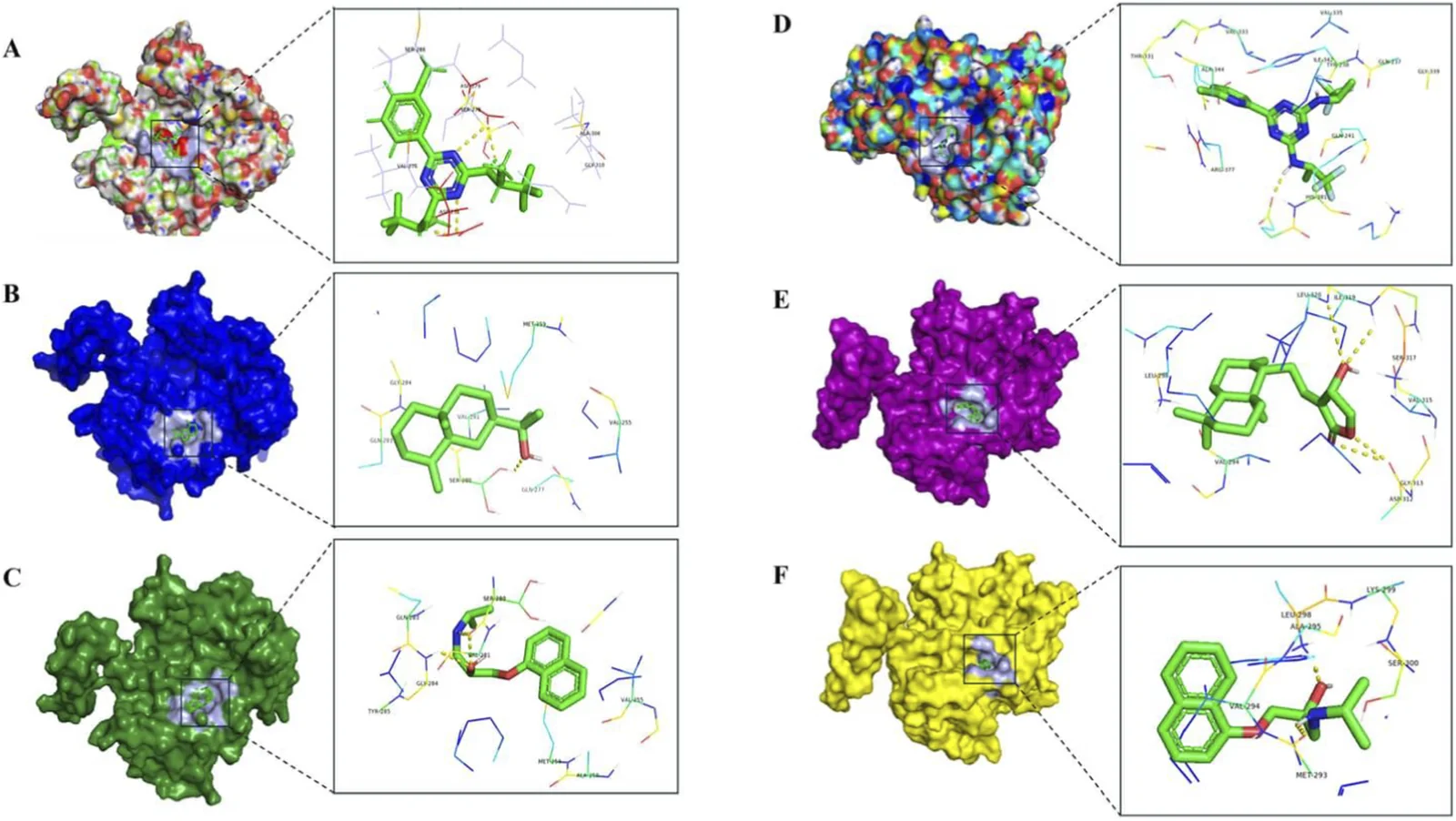
3D visualization of protein-ligand binding interactions of IDH1(left panel) **(A)** Standard, **(B)** I1Hit1, and **(C)** I1Hit2, and IDH2 (right panel) **(D)** Standard, **(E)** I2Hit1, and **(F)** I2Hit2.

The top 20 compounds, including the reference standard, were selected for molecular docking studies, which is a key technique in the drug discovery process. Protein-ligand interactions were evaluated by analysing binding energies, conformational adaptations, and molecular interaction profiles. The top-ranked 20 compounds, including standard, show binding scores ranging between −6.17 kcal/mol to −9.78 kcal/mol against IDH1 and -6.17 kcal/mol to −11.23 kcal/mol against IDH2. Further in comparison with the standard binding affinity of −6.17 kcal/mol with a higher Ki value of 30.15 μM, I1Hit1 and I1Hit2 were found as lead compounds with the strongest binding affinity of −7.57 kcal/mol and −6.82 kcal/mol with lower Ki values of 2.84 μM and 10.08 μM respectively, against IDH1. However, I2Hit1 and I2Hit2 were found as lead compound with strongest binding affinity −11.23 kcal/mol and −7.87 kcal/mol with lower Ki values of 0.00582 μM (5.82 nM) and 1.71 μM respectively, than the standard with lower binding affinity of −6.17 kcal/mol and higher Ki value of 30.2 μM, against IDH2 based on best binding score, conformations, hydrogen bonding, electrostatic force, van der Waals force and other interaction. The 2D interaction of binding sites is visualised and depicted in Figure 2 by using Discovery Studio for both proteins. The bond interactions of standard include three hydrogen bonds formed at TYR272, ASP275, ASP279 residues of bond distance 5.74, 3.67, 4.38 Å, one hydrophobic bond formed at VAL276 of bond distance 5.23 Å and seven other interaction of van der Waals interaction including GLY310, HIS309, ARG109, SER278, LEU288, SER280, GLN283 residues, the lead compounds I1Hit1 forms one hydrogen bond at GLN277 of bond distance 4.04 Å, one carbon-hydrogen (C-H) bond formed at SER280 of bond distance 3.94 Å, eight hydrophobic bonds at LEU120, TRP124, VAL121, ALA258, MET259, VAL281, TRP267, VAL255 of bond distance 4.86, 7.14, 5.36, 4.65, 5.73, 4.59, 6.51, 4.16 Å and two other interactions of van der Waals interaction including residues GLY284, GLN283 and I1Hit2 forms two hydrogen bond at GLY284, SER280 of bond distance 3.00, 3.70 Å, three hydrophobic bonds formed at VAL255, ALA258, MET 259 of bond distance 5.31, 5.77, 4.95 Å and seven other interactions of van der Waals interaction including residues LEU120, VAL121, GLN277, TRP267, TRP124, VAL281, GLN283 against IDH1. In contrast for IDH2 the bond interactions of standard forms two hydrogen bonds at GLU380, HIS381 residues of bond distance 4.07, 4.71 Å, six hydrophobic bonds at ALA344, TYR238, VAL335, ILE342, LYS384, VAL333 of bond distance 4.49, 4.53, 5.08, 6.86, 4.70, 7.05 Å and six other interactions of van der Waals interactions including residues ALA374, SER332, GLU345, THR331, GLU150, LYS242, whereas lead compound I2Hit1 forms of two hydrogen bonds VAL 315, LEU 320 residues of bond distance 4.46, 4.00 Å, two hydrophobic bonds at TRP164, ILE168 of bond distance 7.47, 5.51 Å and eleven other interactions of van der Waals including ASP318, ILE319, SER317, GLY313, ASP312, ILE170, VAL294, PHE304, SER300, LEU298, MET293 and I2Hit2 forms three hydrogen bond network of LEU298, SER300, MET293 residues at bond distance 5.56, 2.71, 4.18 Å, three hydrophobic bonds of LYS299, LEU320, PHE304 residues at bond distance 5.37, 5.76, 6.38 Å and seven other interactions of van der Waals including ILE168, ILE170, ILE319, VAL294, TRP164, GLY302, ALA295, listed in Tables 4, 5 for both the proteins. The 3D visualisation of protein-ligand interactions for both proteins is illustrated in Figure 3 using PYMOL.

**TABLE 4:** Binding affinity, Inhibitory constant (Ki), Interactions and bond distance of the standard and top hit compounds against IDH1.

S.no	PubChem ID	Compound name	Binding energy (kcal/mol)	Inhibitory constant Ki (µM)	Interactions	Bond distance (Å)
1	117817422	Vorasidenib (Standard)	−6.17	30.15	H bonds: TYR272, ASP275, ASP279 Hydrophobic bond: VAL276 van der Waals: GLY310, HIS309, ARG109, SER278, LEU288, SER280, GLN283.	H bonds: 5.74, 3.67, 4.38 Hydrophobic bond: 5.23
2	91457	β-Eudesmol (I1Hit1)	−7.57	2.84	H bond: GLN277 C-H bond: SER280 Hydrophobic bonds: LEU120 TRP124, VAL121, ALA258, MET259, VAL281, TRP267, VAL255 van der Waals: GLY284, GLN283	H-bond: 4.04 C-H bond: 3.94 Hydrophobic bonds: 4.86, 7.14, 5.36, 4.65, 5.73, 4.59, 6.51, 4.16
3	4946	Propranolol (I1Hit2)	−6.82	10.08	H-bonds: GLY284, SER280 Hydrophobic bonds: VAL255, ALA258, MET259. van der Waals: LEU120, VAL121, GLN277, TRP267, TRP124, VAL281, GLN283	H-bond: 3.00, 3.70 Hydrophobic bonds: 5.31, 5.77, 4.95

**TABLE 5:** Binding affinity, Inhibitory constant (Ki), Interactions, and bond distance of the standard and top hit compounds against IDH2.

S.no	Compound ID	Compound name	Binding energy (kcal/mol)	Inhibitory constant Ki (µM)	Interactions	Bond distance (Å)
1	117817422	Vorasidenib (Standard)	−6.17	30.2	H bonds: GLU380, HIS381 Hydrophobic bonds: ALA344, TYR238, VAL335, ILE342, LYS384, VAL333 van der Waals: ALA374, SER332, GLU345, THR331, GLU150, LYS242	H-bond: 4.07, 4.71 Hydrophobic bonds: 4.49, 4.53, 5.08, 6.86, 4.70, 7.05
2	71550939	Isocoronarin D (I2Hit1)	−11.23	0.00582	H bonds: VAL315, LEU320 Hydrophobic bonds: TRP164, ILE168 van der Waals: ASP318, ILE319, SER317, GLY313, ASP312, ILE170, VAL294, PHE304, SER300, LEU298, MET293	H-bond: 4.46, 4.00 Hydrophobic bonds: 7.47, 5.51
3	4946	Propranolol (I2Hit2)	−7.87	1.71	H- bonds: LEU298, SER300, MET293 Hydrophobic bonds: LYS299, LEU320, PHE304 van der Waals: ILE168, ILE170, ILE319, VAL294, TRP164, GLY302, ALA295	H-bond: 5.56, 2.71, 4.18 Hydrophobic bonds: 5.37, 5.76, 6.38

### 3.3 Molecular dynamics simulation analysis

Molecular dynamics (MD) simulation is a computational approach used to investigate the structural dynamics and stability of biomolecular systems, including protein-ligand interactions and to identify ligand-induced conformational changes. Unlike molecular docking, which treats the protein as rigid and ligand as flexible, MD simulations consider the flexibility of both protein and ligand, providing a more realistic representation of molecular behaviour (Ghahremanian et al., 2022; Shah et al., 2024; Sharma and Muniyan, 2024). In our study, MD simulations were performed over a 300 ns trajectory for IDH1, IDH2 and hit compounds (I1Hit1, I1Hit2, I2Hit1 and I2Hit2) to analyse RMSD, RMSF, Rg, SASA, and H-bonds.

#### 3.3.1 Molecular dynamics simulation for IDH1

**FIGURE 4:**
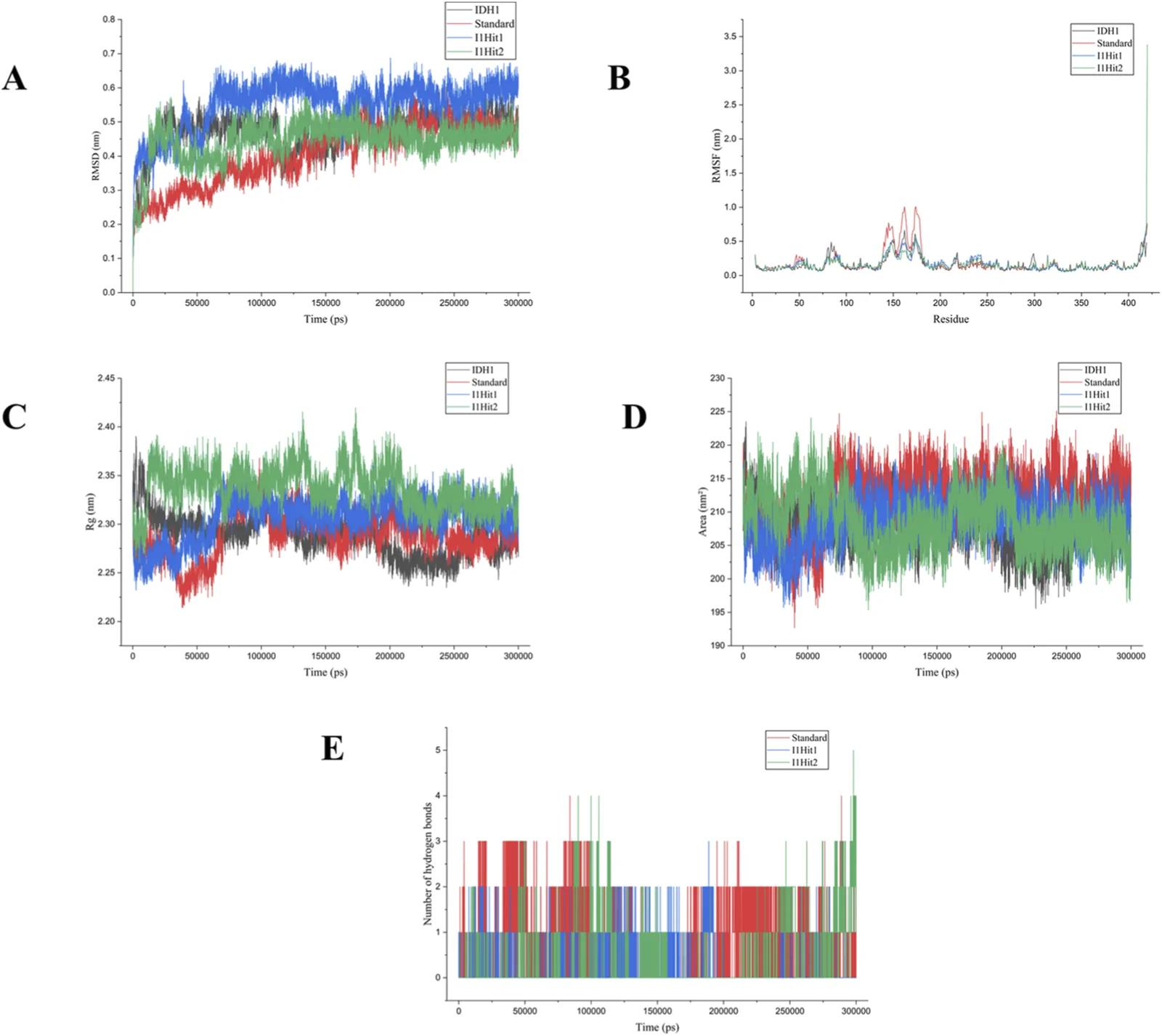
Molecular dynamics simulation analysis of IDH1-ligand complexes: **(A)** RMSD, **(B)** RMSF, **(C)** Rg, **(D)** SASA, **(E)** H-bond.

RMSD estimates ensemble-averaged deviations of protein structure from its reference state and evaluates the stability of the ligand-bound protein complex (Younus et al., 2021). The average RMSD values of IDH1, I1Hit1, and I1Hit2 are 0.47 nm, 0.40 nm, 0.55 nm, and 0.44 nm, respectively. The analysis of the RMSD indicated that the IDH1 and standard displayed higher fluctuation, depicting instability, and had their maximum average deviations increased at 277 ns, 285 ns for IDH1 and 180 ns, 142 ns for standard, respectively. The RMSD of compounds I1Hit1 and I1Hit2 displays less fluctuation, and good stability was observed between 125 and 175 ns for I1Hit2 and 240–300 ns for both I1Hit1 and I1Hit2. RMSF analyse residue-level flexibility during the simulation. The regions of the loop have higher fluctuations, while the secondary structure elements, such as helices and sheets, have lower fluctuations, indicating higher stability of the structure (Gheidari et al., 2024; Sharma and Muniyan, 2024). RMSF calculation revealed that I1Hit1 and I1Hit2 compounds show reduced residue-wise fluctuation with higher stability having low average RMSF values of approximately 0.16 nm and 0.17 nm, respectively than the standard and IDH1. The major fluctuation across 140–170 loop residues was observed for IDH1 and the standard. The average values of the conformational compactness of all complexes were found to be in the range of 2.28–2.33 nm by Rg analysis. The behaviour of I1Hit1 and I1Hit2 was stable in the time range 120–155 ns and 230–280 ns, respectively, with minimal fluctuations. SASA measures the solvent accessible surface area of proteins and monitors structural changes and hydrophobic exposure (Verma et al., 2016; Zhang et al., 2023). The average SASA values of 208.7 nm^2^ for I1Hit1 and 208.5 nm^2^ for I1Hit2, were also lower than the reference standard 211.9 nm^2^, suggesting that these hit compounds are less solvent accessible and have optimal folding. Hydrogen bond analysis during MD simulation can be used to evaluate the stability of protein and protein-ligand interactions (Ghahremanian et al., 2022). This study uncovers underlying binding characteristics, where the average of hydrogen bond counting between the main protease and inhibitors offers measures of interaction stability and dynamics. Hydrogen bond analysis shows I1Hit2 exhibits a higher number of 5 hydrogen bonds, whereas the standard generates up to 4 hydrogen bonds. I1Hit1 and I1Hit2 are consistent with 1 hydrogen bond exhibiting better stability in binding interactions across 120–150 ns, indicating that the consistency of forming interaction was potentially higher. The results of these analyses were plotted and are shown in Figure 4. Overall, MD simulations indicate that I1Hit1 and I1Hit2 display enhanced dynamic properties and structural stability, surpassing the standard compound across multiple parameters.

#### 3.3.2 Molecular dynamics simulation for IDH2

**FIGURE 5:**
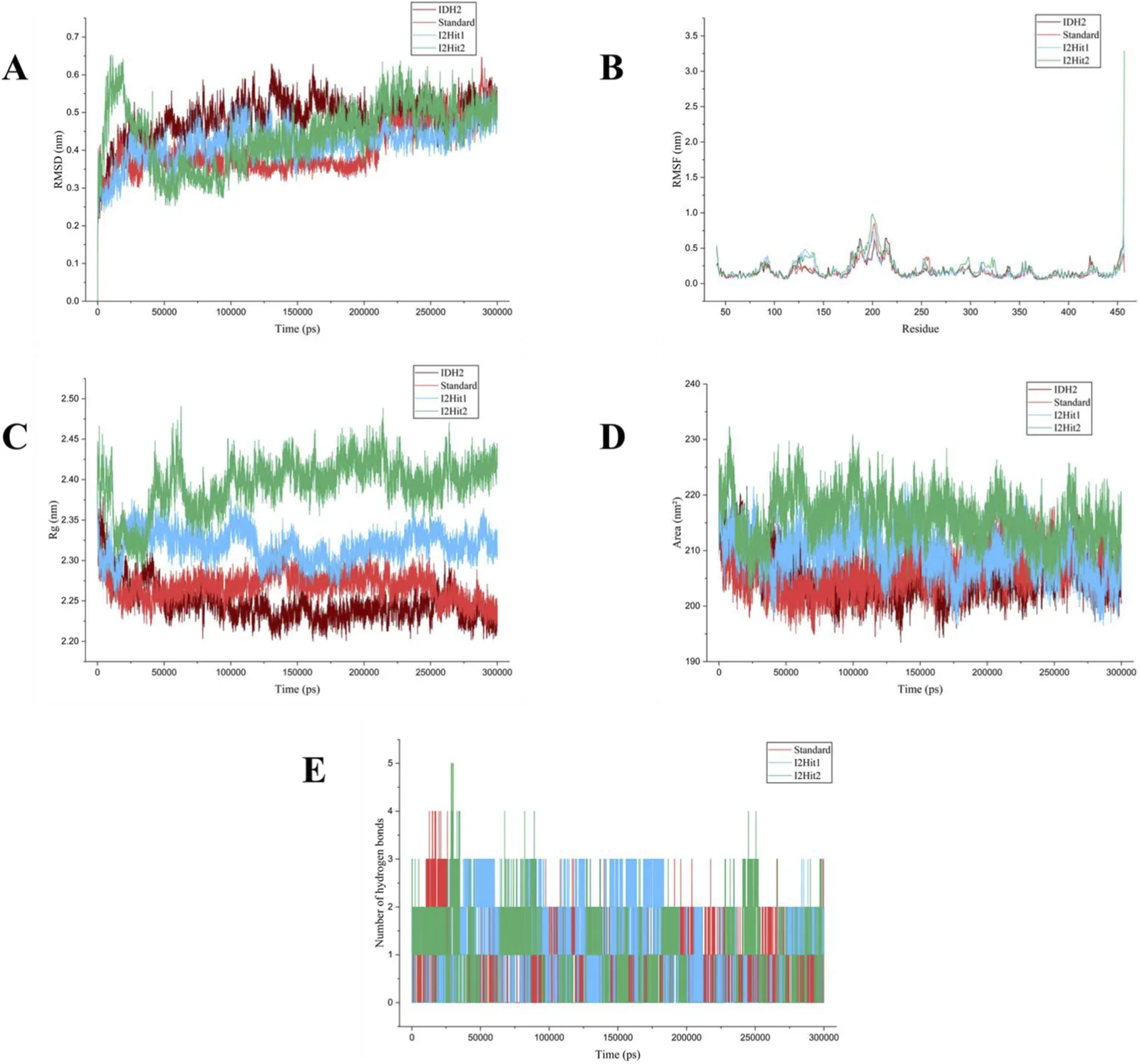
Molecular dynamics simulation analysis of IDH2-ligand complexes: **(A)** RMSD, **(B)** RMSF, **(C)** Rg, **(D)** SASA, **(E)** H-bond.

The result of the molecular dynamics simulation showed the average RMSD values of IDH2, standard, I2Hit1 and I2Hit2 were 0.48 nm, 0.40 nm, 0.41 nm, and 0.44 nm, respectively. The I2Hit2 initially exhibited larger structural fluctuation at 6–19 ns, with deviations ranging from 0.60 to 0.65 nm, later throughout the simulation less fluctuations and better stability were observed. In contrast, standard observed a good stability region from 95 ns to 200 ns, further variation was larger with higher fluctuation over the last 90 ns (210 ns – 300 ns), suggesting conformational changes and greater instability. The I2Hit1 complex showed a relatively low RMSD value of 0.29 nm at 29 ns and found consistent stability across 240–300 ns. Average RMSF values of IDH2, standard, I2Hit1 and I2Hit2 were 0.17 nm, 0.17 nm, 0.18 nm and 0.22 nm, respectively. The highest residue-level fluctuation was observed for I2Hit2, with a peak RMSF of 0.98 nm at residue 200, while the standard complex showed a peak of 0.82 nm at residue 201. In contrast, IDH2 and I2Hit1 exhibited lower fluctuations, with minimum RMSF values of 0.63 nm at residue 202 and 0.73 nm at residue 198, respectively, indicating greater structural stability. The values of Rg were measured to be 2.24 nm, 2.26 nm, 2.31 nm and 2.39 nm for IDH2, standard, I2Hit1 and I2Hit2, respectively, suggesting reliable compactness and structural integrity of the systems. The mean SASA values were 205.56 nm^2^ for IDH2, 206.25 nm^2^ for standard, 209.76 nm^2^ for I2Hit1 and 215.92 nm^2^ for I2Hit2. However, I2Hit1 and I2Hit2 have a higher SASA value, and a consistent stability region was observed at 62–98 ns and 130–200 ns, respectively, in comparison to IDH2 and the standard. Hydrogen bond analysis showed that I2Hit2 and the standard complex formed a maximum of 5 and 4 hydrogen bonds, respectively. I2Hit2 maintained consistent interactions with 2 hydrogen bonds during 2–25 ns and 6–65 ns, while the standard complex maintained 1 hydrogen bond during 150–165 ns and 233–253 ns. I2Hit1 formed up to 3 hydrogen bonds and remained consistently at 2 hydrogen bonds, particularly between 272 and 300 ns, indicating stable interactions. These results of RMSD, RMSF, Rg, SASA and H-bonds are represented as a graph in Figure 5.

### 3.4 Principal component analysis (PCA)

**FIGURE 6:**
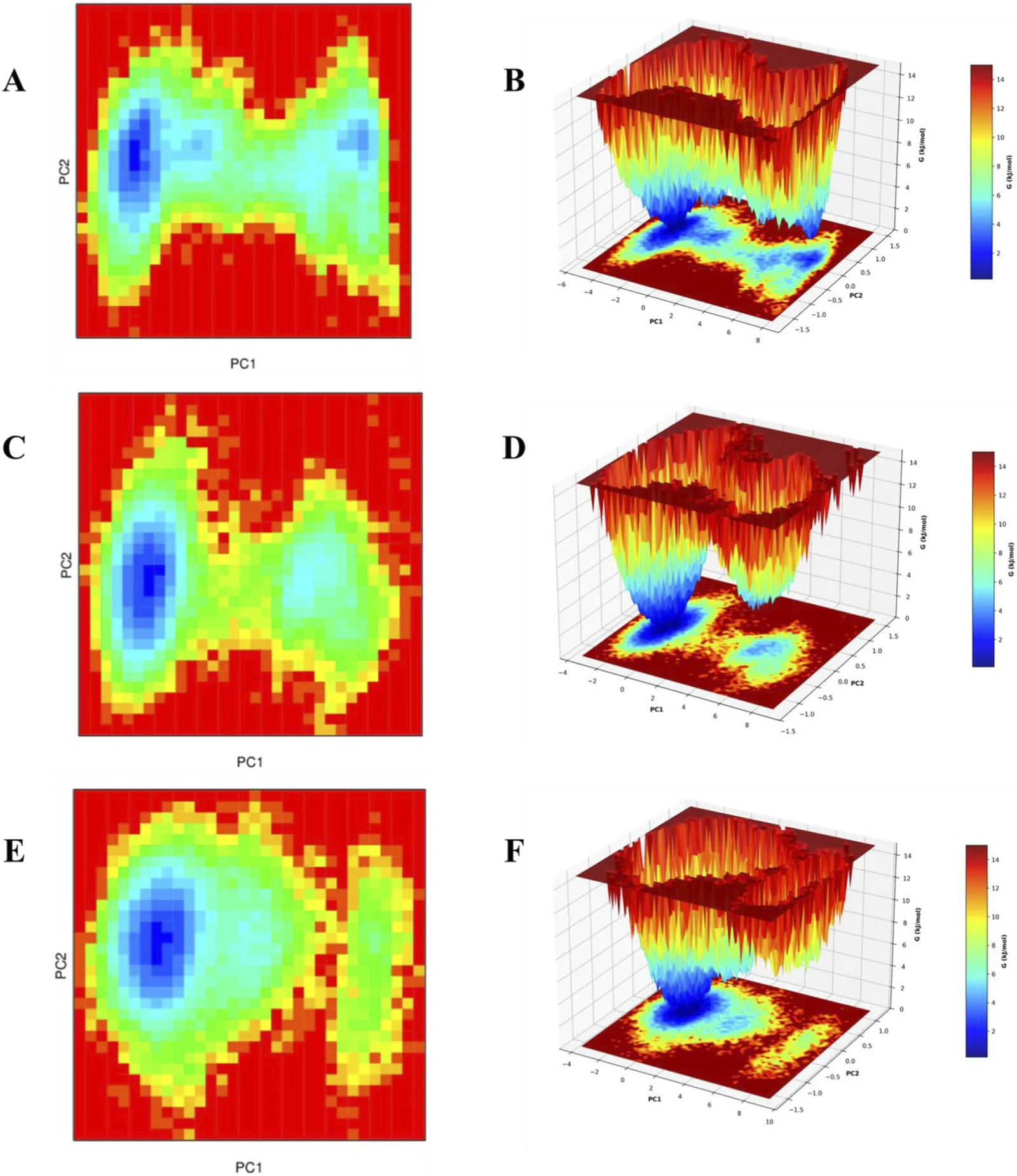
Free energy landscape (FEL) plot between PC1 and PC2. **(A)** 2-D plot of Standard, **(B)** 3-D plot of Standard, **(C)** 2-D plot of I1Hit1, **(D)** 3-D plot of I1Hit1, **(E)** 2-D plot of I1Hit2, **(F)** 3-D plot of I1Hit2.

**FIGURE 7:**
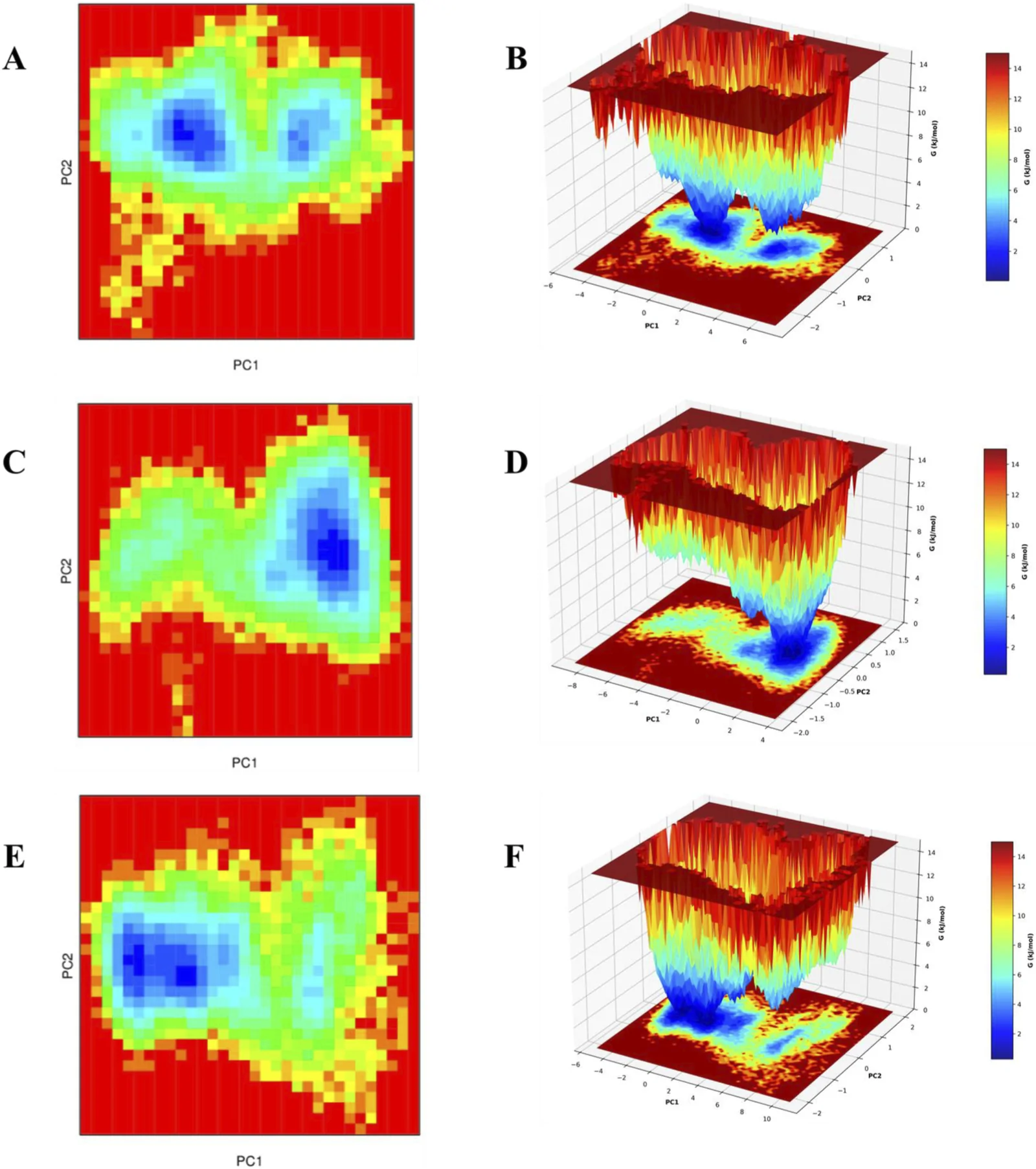
Free energy landscape (FEL) plot between PC1 and PC2. **(A)** 2-D plot of Standard, **(B)** 3-D plot of Standard, **(C)** 2-D plot of I2Hit1, **(D)** 3-D plot of I2Hit1, **(E)** 2-D plot of I2Hit2, **(F)** 3-D plot of I2Hit2.

PCA was conducted to describe the conformational changes in the protein caused by compound binding and to elucidate the dominant collective motions that existed in the MD simulations (Ali et al., 2023). A lower eigenvalue and higher eigenvector typically signify decreased dynamism and increased structural stability. As shown in Supplementary Figure S2, the I1Hit1 complex showed a lower eigenvalue than the standard complex, indicating a less dynamic nature with minimal fluctuations. However, the I2Hit1 complex exhibits a lower eigenvalue than the I2Hit2 complex against IDH2. The PCA plots, with PC1 (x-axis) and PC2 (y-axis), describe the global motion of Cα atoms. Two major clusters are evident along PC1 in both complexes, suggesting that at least two different conformations were sampled during the MD simulations. The IDH1 and standard complexes exhibited wider, more dispersed distributions with lower probabilities, consistent with less stability and greater conformational flexibility. In contrast, I1Hit1 and I1Hit2 generated clear distinct clusters indicating well-defined transitions in Supplementary Figure S3. Similarly, Supplementary Figure S4, shows that the IDH2 and standard complexes exhibit broader conformational distribution, indicating instability and higher conformational space, whereas I2Hit1 and I2Hit2 show denser clustering patterns, suggesting comparatively stable conformational behaviour. Free Energy Landscape (FEL) analysis further supported these findings, where darker blue regions (0–2 kJ/mol) represent the most stable conformers with narrow and wide minima basins. Narrow and wide energy-minimum basins can be distinguished using a 3D FEL plot, a narrow basin often represents more stable conformations (Dubey et al., 2025a). FEL plots generated over 300 ns MD simulations for all complexes revealed Gibbs free energies of 16.4 kJ/mol, 16.9 kJ/mol, and 16.4 kJ/mol for the standard, I1Hit1, and I1Hit2 of IDH1 complexes, respectively, and 16.6 kJ/mol, 16.3 kJ/mol, and 15.9 kJ/mol for the standard, I2Hit1, and I2Hit2 of IDH2 complexes. The standard IDH1 complex exhibits a wider energy basin, suggesting weaker conformational stability, whereas I1Hit1 and I1Hit2 showed narrow, canonical-shaped energy minima with prominent blue regions, indicating the formation of stable conformers. Similarly, standard and I2Hit2 demonstrated prolonged narrow energy regions with consistent blue regions, while the I2Hit1 of the IDH2 complex displayed a single narrow canonical-shaped energy minimum. The 2D and 3D FEL projections for both IDH1 and IDH2 complexes are depicted in Figures 6, 7 respectively.

### 3.5 Binding free energy calculation

**FIGURE 8:**
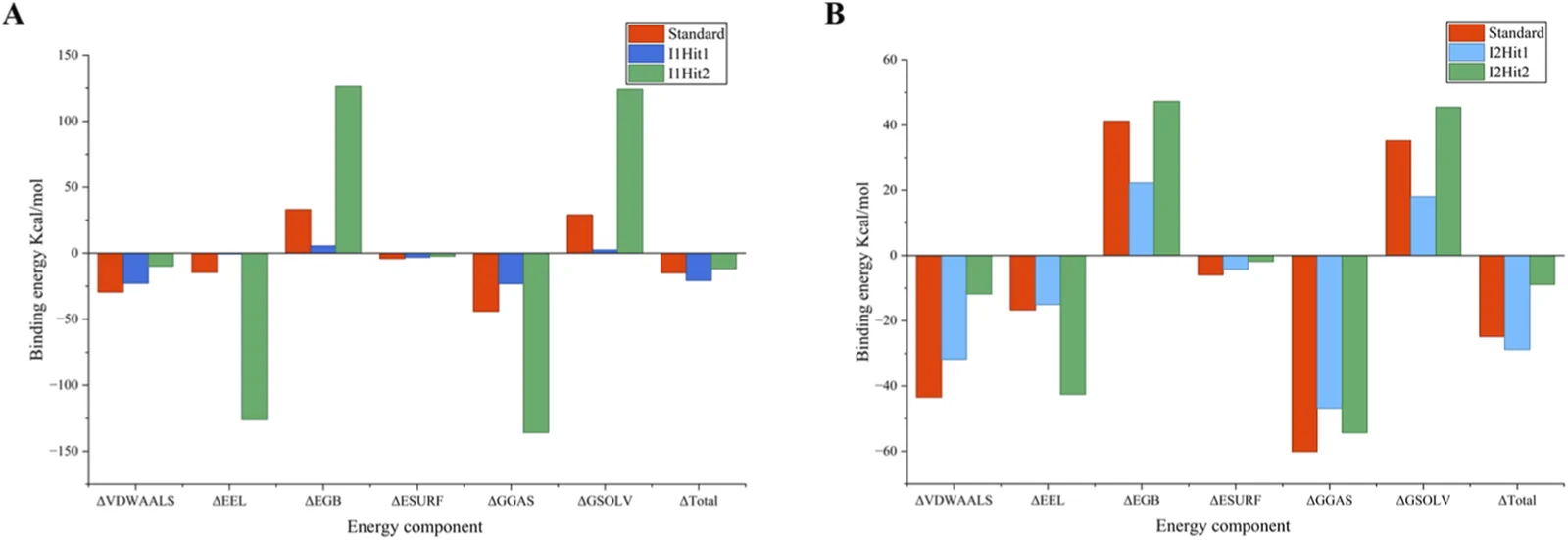
Binding free energy calculation graph, **(A)** Plot for IDH1, **(B)** Plot for IDH2.

Binding free energy calculations provide a quantitative measure of ligand binding affinity within the target site. This approach is widely used in post-docking analysis to allow a deeper understanding of molecular interactions and evaluate the stability of ligand-receptor complexes (Suleman et al., 2022). The result of MMPBSA includes different entropy energies like van der Waals energy (ΔVDWAALS), electrostatic energy (ΔEEL), polar solvation free energy (ΔEGB), and non-polar solvation free energy (ΔESURF), solvation energy (ΔGSOLV), gas face free energy (ΔGGAS), and total binding energy (ΔTotal or ΔG bind). As shown in Table 6 **,** in consideration with IDH1 protein, the ΔVDWAALS for standard, I1Hit1, I1Hit2 was estimated as −29.49 ± 3.30 kcal/mol, −22.75 ± 2.15 kcal/mol, and −9.77 ± 5.01 kcal/mol, on other hand ΔEEL was calculated as −14.60 ± 6.38 kcal/mol, −0.44 ± 1.52 kcal/mol, −126.08 ± 35.07 kcal/mol for standard, I1Hit1, and I1Hit2. The polar contribution(ΔEGB) and non-polar contribution(ΔESURF) for standard, I1Hit1, and I1Hit2 were recorded as 33.11 ± 5.21 and −3.99 ± 0.45 kcal/mol, 5.53 ± 1.53 and −3.01 ± 0.25 kcal/mol, 126.30 ± 30.70 and −2.21 ± 0.60 kcal/mol respectively. The ΔGGAS and ΔGSOLV were determined as −44.09 ± 7.91 kcal/mol and 29.12 ± 4.96 kcal/mol for standard, −23.19 ± 2.50 kcal/mol and 2.52 ± 1.49 kcal/mol for I1Hit1, and -135.86 ± 33.39 kcal/mol and 124.09 ± 30.55 kcal/mol for I1Hit2. The total binding energy (ΔG bind) was calculated as −14.96 ± 4.22 kcal/mol, −20.67 ± 2.32 kcal/mol, and −11.77 ± 4.84 kcal/mol for standard, I1Hit1, and I1Hit2, indicating standard has less stability than I1Hit1, where I1Hit1 shows a higher value with a more stable region, whereas I2Hit2 shows nearer and comparable values with better stability. Furthermore, similarly for IDH2 protein ΔVDWAALS, ΔEEL, ΔGGAS, ΔEGB, ΔESURF and ΔGSOLV, values were demonstrated and represented in Table 6. ΔG bind values exhibit −24.87 ± 3.55 kcal/mol, −28.78 ± 2.67 kcal/mol and −8.91 ± 4.74 kcal/mol for standard, I2Hit1 and I2Hit2, which demonstrate that I2Hit1 is highly stable and has a higher affinity for binding with protein than standard and I2Hit2. The binding free energy of the energy component graph is depicted in Figure 8.

**TABLE 6:** MMPBSA/Binding free energy (Kcal/mol) calculations of protein–ligand complexes.

Protein	Ligand	ΔVDWAALS	ΔEEL	ΔEGB	ΔESURF	ΔGGAS	ΔGSOLV	ΔTotal (kcal/mol)
IDH 1	Standard	−29.49 ± 3.30	−14.60 ± 6.38	33.11 ± 5.21	−3.99 ± 0.45	−44.09 ± 7.91	29.12 ± 4.96	−14.96 ± 4.22
	I1Hit1	−22.75 ± 2.15	−0.44 ± 1.52	5.53 ± 1.53	−3.01 ± 0.25	−23.19 ± 2.50	2.52 ± 1.49	−20.67 ± 2.32
	I1Hit2	−9.77 ± 5.01	−126.08 ± 35.07	126.30 ± 30.70	−2.21 ± 0.60	−135.86 ± 33.39	124.09 ± 30.55	−11.77 ± 4.84
IDH 2	Standard	−43.42 ± 2.75	−16.69 ± 3.44	41.22 ± 3.00	−5.98 ± 0.34	−60.11 ± 4.22	35.24 ± 2.95	−24.87 ± 3.55
	I2Hit1	−31.78 ± 2.66	−15.02 ± 3.23	22.21 ± 2.42	−4.19 ± 0.31	−46.80 ± 3.56	18.02 ± 2.33	−28.78 ± 2.67
	I2Hit2	−11.79 ± 3.50	−42.56 ± 25.10	47.22 ± 21.40	−1.79 ± 0.40	−54.34 ± 25.11	45.43 ± 21.17	−8.91 ± 4.74

### 3.6 Toxicity prediction for hit compounds

In early stages of drug discovery, computational toxicity predictions serve as a valuable tool for proactively filtering out compounds that are likely to exhibit clinical failure due to toxicological concerns (Cavasotto and Scardino, 2022). Pharmacological activity prediction using the PASS online server revealed a high probability of antineoplastic activity for the selected hit compounds, as indicated by elevated Pa values and correspondingly low Pi values. Specifically, I1Hit1 exhibited a Pa of 0.792 and Pi of 0.013, I2Hit1 showed a Pa of 0.960 and Pi of 0.004, while PubChem ID 4946 (I1Hit2/I2Hit2) demonstrated a Pa of 0.118 and Pi of 0.058. These results are significantly better than the standard values of Pa = 0.521 and Pi = 0.065, indicating strong potential for anticancer activity. Moreover, the compounds were also found to be inducers of TP53 expression. Pa and Pi values of I1Hit1 were recorded as 0.373 and 0.157 respectively. I2Hit1 showed a Pa of 0.520 and Pi of 0.081, and I1Hit2 & I2Hit2 yielded a Pa of 0.463 and Pi of 0.108. These values significantly exceed the standard values of Pa = 0.291 and Pi = 0.218, indicating a potential role in modulating TP53 expression. Collectively, these findings highlight the therapeutic rationale for the hit compounds as cancer therapeutic agents with combined antineoplastic activity and TP53-enhancing properties. Results of this PASS prediction analysis are summarized in Table 7. For *In silico* cytotoxicity prediction, CLC-Pred 2.0, a software tool, was used to predict the cytotoxic activity of chemical compounds on tumour and non-tumour cell lines. The predicted cytotoxicity of the standard and various hit compounds on different brain tumour cell lines has been summarised in Table 8. Although the hit compounds were variable in their predicted activity (Pa) and inactivity (Pi), they consistently had higher Pa values and lower Pi values compared to those of the standard, indicating enhanced cytotoxic efficacy. Moreover, the invariable accuracy prediction (IAP) of all hit compounds has been predicted to 0.8 to 0.9, indicating extremely reliable and accurate predictions with minimal deviation. Overall, these findings highlight the value of all hit compounds as potential lead structures to target brain tumour cell lines with potent cytotoxic activity. Cardiotoxicity prediction using Pred-hERG 5.0 server for the hit compounds suggested differences in their blockage of hERG channel. In the multiclass prediction, standard compound was classified as a moderate blocker, I1Hit1 as weak blocker, I2Hit1 as a non-blocker, and I1Hit2 and I2Hit2 as moderate blocker. The corresponding confiability scores were 95.10 for the standard, 92.14 for I1Hit1, 99.12 for I2Hit2, and 89.96 for I1Hit2 and I2Hit2. Assessment of the applicability domain (AD) in Pred-hERG 5.0 server also revealed that the standard compound is within it, implying possible prediction precision. In the case of I1Hit1 and I2Hit1, both compounds were known to be outside the AD, whereas I1Hit2 and I2Hit2 (PubChem ID 4946) were categorised in the AD. These results indicate that I1Hit2 and I2Hit2 and the standard compound have comparable predictability in terms of cardiotoxicity, as shown below in Table 9. The *in silico* rat acute toxicity evaluation was performed by GUSAR online tool, which provides the prediction of compound toxicities through numerous administrations. Oral administration is currently the most convenient and viable vehicle for drug administration due to its cost-effectiveness and greater patient adherence (Alqahtani et al., 2021). In this context, the standard compound was found to be outside the applicability domain (AD) for intraperitoneal (IP) and oral routes, but within the AD for intravenous (IV) and subcutaneous (SC) administration. By contrast, the AD for all active hits were predicted across all administered routes, implying a wider range of potential drug administration. Accordingly, although vorasidenib (Standard) is clinically administered orally, its predicted toxicity was evaluated across all four routes to enable the consistent comparison with the hit compounds. The resulting LD50 values were further classified according to the standard six-class acute toxicity scale, in which lower class numbers denote higher toxicity (Class 1: LD50 ≤5 mg/kg) and higher class numbers denote lower toxicity (Class 6: LD50 > 5000 mg/kg). I1Hit1 and I2Hit1 were predicted to fall within toxicity class 3–5 across all four administration routes, corresponding to LD50 values ranging from approximately 29,390 mg/kg (IV) to 2,459,000 mg/kg (oral) for I1Hit1, and 7,136 mg/kg (IV) 1,600,000 mg/kg (oral) for I2Hit1, indicating generally low predicted acute toxicity and a favourable safety scale relative to the standard. I1Hit2/I2Hit2 was predicted to fall within Class 4 across all routes, consistent with its established clinical safety profile as an approved therapeutic drug. These findings collectively suggest that the identified hit compounds exhibit favourable predicted acute toxicity profiles with reliable applicability domain coverage across all tested routes, supporting their suitability for further experimental safety evaluation. A summary of the administration route predictions for both the standard and hit compounds is presented in Table 10.

**TABLE 7:** PASS prediction for standard and hit compounds.

Compound & PubChem ID	Vorasidenib Standard (117817422)		β-Eudesmol I1Hit1 (91457)		Isocoronarin D I2Hit1 (71550939)		Propranolol I1Hit2 & I2Hit2 (4946)	
Activity	Pa	Pi	Pa	Pi	Pa	Pi	Pa	Pi
Antineoplastic	0.521	0.065	0.792	0.013	0.960	0.004	0.118	0.058
TP53 expression enhancer	0.291	0.218	0.373	0.157	0.520	0.081	0.463	0.108

**TABLE 8:** Cytotoxicity prediction coefficient with tumour cell lines for standard and hit compounds.

Compound & PubChem ID	Brain tumour	Cell lines	Pa	Pi	IAP*
Vorasidenib Standard (117817422)	Glioblastoma	U-87MG	0.285	0.019	0.921
	Glioblastoma	D54	0.162	0.027	0.995
β-Eudesmol I1Hit1 (91457)	Glioma	C6	0.303	0.026	0.98
	Astrocytoma	SNB-19	0.131	0.093	0.948
Isocoronarin D I2Hit1 (71550939)	Astrocytoma	U-251	0.731	0.005	0.883
	Glioma	C6	0.337	0.021	0.98
	Glioblastoma	SF-539	0.197	0.145	0.888
	Glioblastoma	SF-268	0.189	0.171	0.89
	Astrocytoma	SNB-19	0.127	0.101	0.948
Propranolol I1Hit2 & I2Hit2 (4946)	Glioblastoma	A172	0.385	0.044	0.807
	Glioma	C6	0.354	0.018	0.98
	Glioblastoma	T98G	0.184	0.057	0.912

**TABLE 9:** Cardiotoxicity prediction for standard and hit compounds by using the Pred-hERG server.

Compound & PubChem ID	Multiclass prediction	Confiability %	Applicability domain (AD)
Vorasidenib Standard (117817422)	Moderate blocker	95.1	Inside AD
β-Eudesmol I1Hit1 (91457)	Weak blocker	92.14	Outside AD
Isocoronarin D I2Hit1 (71550939)	Non-blocker	99.12	Outside AD
Propranolol I1Hit2 & I2Hit2 (4946)	Moderate blocker	89.96	Inside AD

**TABLE 10:** Administration route for rat acute toxicity prediction.

Compound	Rat IP			Rat IV			Rat oral			Rat SC		
	LD50 (mg/kg)	Toxicity class		LD50 (mg/kg)	Toxicity class		LD50 (mg/kg)	Toxicity class		LD50 (mg/kg)	Toxicity class	
		Class	In AD/Out AD		Class	In AD/Out AD		Class	In AD/Out AD		Class	In AD/Out AD
Vorasidenib Standard (117817422)	577,100	5	Out AD	82,190	4	In AD	1,518,000	4	Out AD	438,900	4	In AD
β-Eudesmol I1Hit1 (91457)	872,800	5	In AD	29,390	3	In AD	2,459,000	5	In AD	620,500	4	In AD
Isocoronarin D I2Hit1 (71550939)	542,800	5	In AD	7,136	3	In AD	1,600,000	4	In AD	64,490	3	In AD
Propranolol I1Hit2 & I2Hit2 (4946)	197,700	4	In AD	60,300	4	In AD	988,700	4	In AD	849,700	4	In AD

## 4 Discussion

GBM is the most aggressive primary brain tumour, characterized by therapeutic resistance, high recurrence, leading to poor survival outcomes (Markov et al., 2023) and most frequently observed as metabolic dysregulation caused by IDH enzymes. Beyond its direct enzymatic effects, mutant IDH exerts broader influence on glioma biology through interconnected signaling, immune, and metabolic networks that extend well beyond the IDH active site targeted in this study. The accumulation of 2-HG competitively inhibits α-ketoglutarate-dependent dioxygenases, including TET methylcytosine dioxygenases and JmjC domain-containing histone demethylases, resulting in a DNA and histone hypermethylation phenotype that epigenetically reprograms the glioma cell transcriptome (Choate et al., 2024). This epigenetic remodelling extends into immune regulation in IDH-mutated low-grade gliomas, interferon signaling undergoes epigenetic silencing that reduces PD-L1 expression and limits T-cell exhaustion, while loss of the epigenetic regulator ATRX has separately been shown to drive PD-L1 production alongside multiple immunosuppressive cytokines, illustrating that IDH and co-occurring epigenetic alteration collectively modulate the immune landscape of glioma (Wu et al., 2025). This immune remodeling occurs amid the highly immunosuppressive and heterogeneous GBM microenvironment, in which myeloid-derived suppressor cells and tumour-associated macrophages dominate the immune infiltrate, neutrophils within this microenvironment express arginase1 (ARG1), reactive oxygen species (ROS), nitric oxide synthase in the presence of granulocyte colony-stimulating factor (G-CSF) and transforming growth factor-beta (TGF-β), thereby inactivating tumour-infiltrating T cells and reinforcing immune evasion independent of IDH status (Sun et al., 2024). In parallel, IDH-mutant glioma cells exhibit considerable metabolic plasticity, and IDH1-mutant glioma cell lines have been demonstrated to undergo metabolic rewiring in response to glutamine/glutamate pathway inhibition, maintaining 2-HG production through alternative metabolic pathways (Ruiz-Rodado et al., 2020). Collectively, these signaling, immune and metabolic layers indicate that IDH-directed monotherapy, however favourable at the binding level, may be insufficient to overcome the multifactorial resistance mechanisms operating within the GBM microenvironment, underscoring the need for combination therapeutic strategies in future translational work.

In addition, IDH mutations in GBM frequently co-occur with alterations in TP53, ATRX, EGFR, and MGMT, and these co-occurring alterations are not incidental to therapeutic response. IDH1, ATRX, and P53 expression status carry independent clinical and prognostic significance in glioblastoma, MGMT promoter methylation status remains a well-established determinant of response to alkylating chemotherapy, and EGFR, despite being mutationally active in over half of GBM cases, has so far proven difficult to target successfully due to poor tumour penetration and pathway redundancy (Mansour et al., 2025; Weller et al., 2024; Ezzati et al., 2024). Computational screening approaches, included in this study, assess IDH inhibition in isolation, without capturing this genomic complexity, while actual therapeutic response in genomically heterogeneous GBM population is likely to be further modulated by TP53, ATRX, EGFR and MGMT status.

Recent clinical investigations targeting mutant IDH enzymes have provided important insights into glioma therapy and remain challenging in terms of treatment. IDH inhibitors such as Ivosidenib (NCT02073994) of Phase I trials with a median progression-free survival (mPFS) of 13.6 months and Vorasidenib (NCT02481154) have demonstrated promising activity in IDH-mutant gliomas by reducing the 2-HG level and prolonging progression-free survival in selected patient populations (Carosi et al., 2024). Notably, the phase III INDIGO trial (NCT04164901) showed that Vorasidenib significantly improved progression-free survival (PFS) in patients with residual or recurrent grade 2 IDH-mutant glioma, highlighting the therapeutic potential of mutant IDH inhibition in lower-grade disease (Carosi et al., 2024). Another study reported BAY1436032 (NCT02746081) of a phase I trial to target IDH-mutant low-grade glioma, involving objective response rate (ORR) and PFS of up to 30 months (Pusch et al., 2017). A perioperative Phase I trial (NCT03343197) further reports that combined vorasidenib and ivosidenib treatment reduces 2-HG concentrations by approximately 92%, underscoring the functional on-target activity of IDH inhibition in CNS and Olutasidenib (NCT03684811) achieved a disease control rate of only 48% in relapsed IDH1-mutant solid tumours (Carosi et al., 2024). Beyond IDH-targeted therapies, the broader landscape of GBM clinical trials underscores the profound translational challenges inherent to this disease. Phase II evaluation of BGJ398 (Infigratinib, NCT01975701) in recurrent GBM demonstrated only 6 months of PFS (Lassman et al., 2022). Ongoing trials including NEO212 (NCT06047379) targeting IDH-mutant astrocytoma and GBM, Safusidenib (NCT05303519, NCT04458272) in IDH-mutant grade 2–4 glioma (Lin et al., 2024; Natsume et al., 2023), and DS-1001b (NCT03030066) in IDH1-R132 mutations, underscore ongoing efforts to overcome barriers and act as efficient inhibitors (Natsume et al., 2023). Bevacizumab-based combinations (NCT05201326) have similarly yielded limited survival benefit, reflecting the persistent challenge of translating encouraging phase II outcomes into successful phase III trials in GBM. However, despite these advances and the efficacy of IDH inhibitors in high-grade gliomas and GBM, critical limitations include tumour heterogeneity, acquired resistance mechanisms, BBB penetration and the complex immunometabolic landscape of advanced tumours. Such outcomes highlight the translational gap between computational or experimental predictions and clinical efficacy in glioma treatment. These challenges highlight the need for effective therapies capable of crossing the BBB and targeting tumour progression (Chakravarty et al., 2021).

Our study proposes an integrated *in silico* strategy for glioblastoma therapy in targeting IDH enzymes, involving virtual screening, pharmacokinetic and ADME profiling, toxicity evaluation, molecular docking and MD simulations. Post-simulation analyses, including RMSD, RMSF, Rg, SASA, Hydrogen bonding, PCA, FEL, and MMPBSA, were employed to assess the stability, binding affinity, and dynamic behaviour of candidate compounds. In our study, standard and hit compounds act as better computationally predicted inhibitors with improved binding affinities for both IDH1 and IDH2, with interactions involving key residues such as TRP124, VAL 255, MET259, TYR272, GLN 277, and SER280 against IDH1, TRP164, LEU 298, ASP312 and LEU 320 against IDH2, than the study reported by (Balaji et al., 2024) We compared our results with previously reported IDH1/IDH2 inhibitor studies (Balaji et al., 2024), in which the standard compound Vorasidenib exhibited binding affinities of −5.47 kcal/mol against IDH1 and -6.27 kcal/mol against IDH2, indicating comparatively stronger interaction with IDH2 than IDH1. In contrast, our analysis revealed improved standard binding affinity of −6.17 kcal/mol against both IDH1 and IDH2, with our hit compounds I1Hit1 (−7.57 kcal/mol) and I2Hit1 (−11.23 kcal/mol) exhibiting improved predicted binding affinity relative to the standard. Post-simulation analyses indicated favourable dynamic stability for the lead complexes relative to their respective native proteins, with I1Hit1 and I2Hit1 showing consistently lower RMSF values, narrower and well-defined energy basins in PCA/FEL analysis, and persistent hydrogen bonding, supporting stable and reproducible binding conformations throughout the simulation trajectories. Binding free energy calculations using the MM-PBSA method further distinguished the relative affinities of the compounds. The I1Hit1 displayed strong binding of −20.67 ± 2.32 kcal/mol, whereas I1Hit2 showed −11.77 ± 4.84 kcal/mol with more reliable interactions with the standard (−14.96 ± 4.22 kcal/mol) against IDH1. For IDH2 complexes, I2Hit1 achieved the most favourable binding energy of −28.78 ± 2.67 kcal/mol, compared to I2Hit2 and the standard with binding energies of −8.91 ± 4.74 kcal/mol and −24.87 ± 3.55 kcal/mol, respectively. Collectively, in our study, I1Hit1 and I2Hit1 were identified as promising hit compounds against mutant IDH targets based on integrated *in silico* analyses. In our study, vorasidenib exhibited binding free energies of −14.96 ± 4.22 kcal/mol against mutant IDH1 and -24.87 ± 3.55 kcal/mol against mutant IDH2. Similar analyses of MM-PBSA were performed with different isoforms of mutant IDH and reported as a separate study examining the mechanistic role of olutasidenib in the selective inhibition of mutated IDH1 with a total binding free energy of −56.82 kcal/mol against mutant IDH1, considerably higher than the corresponding value of −14.48 kcal/mol, against mutant IDH2 (Salifu et al., 2022). Interestingly, I1Hit2 & I2Hit2 (PubChem ID 4946), identified as a potential hit compound in the present study, have been previously reported to inhibit GBM cell proliferation through Notch1 and Hes1 signaling pathway *in vitro*. While distinct from IDH-mediated mechanisms, this evidence reinforces its potential as a multi-target agent in GBM therapy (Kim et al., 2021). Notably, Propranolol (I1Hit2 & I2Hit2, PubChem ID 4946), a clinically approved cardiovascular beta-blocker that was independently identified as a top computational hit against both mutant IDH1 and IDH2, represents a drug repurposing finding of translational significance. Its established safety profile, oral bioavailability, predicted BBB permeability, and commercial availability make it a promising candidate for further experimental investigation in IDH-mutant GBM, consistent with emerging evidence supporting the anticancer potential of beta-adrenergic receptor antagonists in glioma. Although I1Hit1 has been reported to exert anti-tumour effects through inhibition of tumour angiogenesis and endothelial proliferation (Ma et al., 2008), and I2Hit1 has been reported for diverse biological activities, including cytotoxic and anti-inflammatory properties, their potential role in targeting IDH-driven oncogenic metabolism in glioblastoma remains largely unexplored. In this regard, I1Hit1 (PubChem ID 91457) and I2Hit1 (PubChem ID 71550939) are identified by our docking, molecular dynamics simulation, PCA, and MMPBSA analyses as novel small-molecule inhibitors that may effectively modulate IDH-driven oncogenic metabolism in GBM. Importantly, their predicted pharmacokinetic and toxicity profiles further support their efficiency as lead compounds. The present study employed a multi-stage orthogonal computational validation comprising molecular docking, 300 ns of MDS, PCA, MM-PBSA calculations and pharmacophore analysis for a potential drug candidate with high binding affinity and stability to IDH1 and IDH2. Nevertheless, these promising compounds required further experimental validation including, recombinant IDH-R132H and IDH2-R140Q enzymatic inhibition assays, 2-HG quantification and cytotoxicity evaluation in GBM cell lines (Josephine et al., 2026; Schulz et al., 2022). Consequently, experimental investigation is crucial for establishing their therapeutic potential and advancing them toward clinical translation.

## 5 Conclusion

This study employed an integrated computational approach to identify potential inhibitors targeting mutant IDH1 and IDH2 enzymes in GBM. PubChem ID 91457 (I1Hit1) against IDH1 and PubChem ID 71550939 (I2Hit1) against IDH2 were identified as individual potential hit compounds following multiple virtual screening, ADMET filtering, and several molecular analyses. These compounds demonstrated better binding affinity, favourable drug-likeness, and enhanced structural stability compared to the standard reference compound. Further, molecular docking, molecular dynamics simulations, PCA, and MMPBSA analyses supported the stability and interaction strength of these complexes, suggesting their potential to inhibit both cytosolic and mitochondrial IDH isoforms. The identified hit compounds may regulate IDH-associated metabolic alterations, including redox imbalance, NADPH depletion, oxidative stress, and epigenetic dysregulation associated with GBM progression. Collectively, these findings highlight I1Hit1 and I2Hit1 as computationally prioritized candidates for IDH-targeted therapy in GBM, and the integrated computational framework outlined in this study provides a replicable platform for advancing novel IDH-targeted inhibitors toward preclinical investigation. As this study is purely computational in scope, future experimental validation, including enzymatic inhibition assays, 2-HG quantification, and cell-based evaluation in IDH-mutant GBM cell lines, is essential to confirm the therapeutic potential of these identified candidates and advance them toward clinical translation.

## Data Availability

The original contributions presented in the study are included in the article/Supplementary Material, further inquiries can be directed to the corresponding author.
