# Supplementary material for: Targeting IDH-driven Oncometabolism in glioblastoma: a computational approach for the identification of potential inhibitors
Source: Front Bioinform. 2026 Aug 26;6:1884553. doi: 10.3389/fbinf.2026.1884553 (PMC13553832; doi:10.3389/fbinf.2026.1884553)
Supplement: Supplementary file 1 [file Supplementaryfile1.docx]

**Supplementary Table 1 (ST1):** Screening studies of compounds by ADME properties

| **S.no** | **PubChem ID** | **Lipinski rule of five** | **BBB permeant** | **PAINS** | **Bioavailability score** | **GI absorption** |
| --- | --- | --- | --- | --- | --- | --- |
| 1 | Standard (117817422) | Yes | Yes | 0 | 0.55 | High |
| 2 | 11102092 | Yes | Yes | 0 | 0.55 | High |
| 3 | 5471228 | Yes | Yes | 0 | 0.55 | High |
| 4 | 71550939 | Yes | Yes | 0 | 0.55 | High |
| 5 | 91457 | Yes | Yes | 0 | 0.55 | High |
| 6 | 91895315 | Yes | Yes | 0 | 0.55 | High |
| 7 | 16085188 | Yes | Yes | 0 | 0.55 | High |
| 8 | 120706 | Yes | Yes | 0 | 0.55 | High |
| 9 | 10086184 | Yes | Yes | 0 | 0.55 | High |
| 10 | 929262 | Yes | Yes | 0 | 0.55 | High |
| 11 | 121231417 | Yes | Yes | 0 | 0.55 | High |
| 12 | 3616 | Yes | Yes | 0 | 0.55 | High |
| 13 | 91970648 | Yes | Yes | 0 | 0.55 | High |
| 14 | 72937507 | Yes | Yes | 0 | 0.55 | High |
| 15 | 4171 | Yes | Yes | 0 | 0.55 | High |
| 16 | 4946 | Yes | Yes | 0 | 0.55 | High |
| 17 | 220401 | Yes | Yes | 0 | 0.55 | High |
| 18 | 77282689 | Yes | Yes | 0 | 0.55 | High |
| 19 | 13816761 | Yes | Yes | 0 | 0.55 | High |
| 20 | 62907 | Yes | Yes | 0 | 0.55 | High |

**Supplementary Table 2 (ST2):** Comprehensive molecular properties and Drug-Likeness evaluation for screened compounds

| **S.no** | **PubChem ID** | **Molecular weight** | **HBD** | **HBA** | **Log p** | **TPSA** | **Rotatable bond count** | **Ghose** | **Veber** | **Egan** | **Muegge** |
| --- | --- | --- | --- | --- | --- | --- | --- | --- | --- | --- | --- |
| 1 | Standard (117817422) | 414.74 | 2 | 10 | 5.29 | 75.62 | 7 | 1 | 0 | 1 | 1 |
| 2 | 11102092 | 432.51 | 1 | 7 | 4.03 | 75.61 | 6 | 0 | 0 | 0 | 0 |
| 3 | 5471228 | 308.5 | 2 | 2 | 5.1 | 40.46 | 4 | 0 | 0 | 0 | 1 |
| 4 | 71550939 | 318.45 | 1 | 3 | 4.36 | 46.53 | 2 | 0 | 0 | 0 | 0 |
| 5 | 91457 | 222.37 | 1 | 1 | 3.74 | 20.23 | 1 | 0 | 0 | 0 | 1 |
| 6 | 91895315 | 318.45 | 3 | 3 | 2.94 | 60.69 | 2 | 0 | 0 | 0 | 0 |
| 7 | 16085188 | 238.37 | 1 | 2 | 2.81 | 29.46 | 1 | 0 | 0 | 0 | 0 |
| 8 | 120706 | 357.49 | 2 | 4 | 1.1 | 57.61 | 1 | 0 | 0 | 0 | 0 |
| 9 | 10086184 | 300.39 | 1 | 3 | 2.78 | 46.53 | 0 | 0 | 0 | 0 | 0 |
| 10 | 929262 | 250.38 | 0 | 2 | 4.32 | 26.3 | 0 | 0 | 0 | 0 | 0 |
| 11 | 121231417 | 312.41 | 2 | 3 | 3.15 | 64.35 | 6 | 0 | 0 | 0 | 0 |
| 12 | 3616 | 200.28 | 2 | 2 | 0.28 | 58.2 | 9 | 0 | 0 | 0 | 0 |
| 13 | 91970648 | 276.42 | 2 | 2 | 2.63 | 41.13 | 8 | 0 | 0 | 0 | 0 |
| 14 | 72937507 | 334.45 | 1 | 2 | 3.5 | 46.33 | 5 | 0 | 0 | 0 | 0 |
| 15 | 4171 | 267.36 | 2 | 4 | 1.88 | 50.72 | 9 | 0 | 0 | 0 | 0 |
| 16 | 4946 | 259.34 | 2 | 3 | 2.98 | 41.49 | 6 | 0 | 0 | 0 | 0 |
| 17 | 220401 | 371.43 | 1 | 6 | 1.37 | 66.02 | 5 | 0 | 0 | 0 | 0 |
| 18 | 77282689 | 357.49 | 2 | 4 | 1.1 | 60.77 | 1 | 0 | 0 | 0 | 0 |
| 19 | 13816761 | 306.48 | 2 | 2 | 4.89 | 40.46 | 1 | 0 | 0 | 0 | 0 |
| 20 | 62907 | 211.34 | 1 | 1 | 3.47 | 29.1 | 4 | 0 | 0 | 0 | 0 |

**Supplementary Table 3 (ST3):** Predicted Toxicity profile for screened compounds

| **S.no** | **PubChem ID** | **Mutagenicity** | **Carcinogenicity** | **Toxicophore** | **H-HT** | **DILI** |
| --- | --- | --- | --- | --- | --- | --- |
| 1 | Standard (117817422) | 0.009 | 0.199 | 0 | 0.99 | 0.916 |
| 2 | 11102092 | 0.068 | 0.032 | 0 | 0.053 | 0.257 |
| 3 | 5471228 | 0.002 | 0.035 | 0 | 0.052 | 0.019 |
| 4 | 71550939 | 0.123 | 0.292 | 0 | 0.023 | 0.166 |
| 5 | 91457 | 0.009 | 0.069 | 0 | 0.047 | 0.087 |
| 6 | 91895315 | 0.01 | 0.032 | 0 | 0.05 | 0.036 |
| 7 | 16085188 | 0.01 | 0.206 | 0 | 0.383 | 0.223 |
| 8 | 120706 | 0.01 | 0.228 | 0 | 0.228 | 0.041 |
| 9 | 10086184 | 0.092 | 0.121 | 0 | 0.229 | 0.093 |
| 10 | 929262 | 0.006 | 0.249 | 0 | 0.136 | 0.134 |
| 11 | 121231417 | 0.138 | 0.036 | 0 | 0.384 | 0.129 |
| 12 | 3616 | 0.1 | 0.012 | 0 | 0.113 | 0.044 |
| 13 | 91970648 | 0.009 | 0.031 | 0 | 0.17 | 0.031 |
| 14 | 72937507 | 0.021 | 0.152 | 0 | 0.391 | 0.038 |
| 15 | 4171 | 0.055 | 0.093 | 0 | 0.339 | 0.007 |
| 16 | 4946 | 0.064 | 0.155 | 0 | 0.115 | 0.017 |
| 17 | 220401 | 0.227 | 0.034 | 0 | 0.178 | 0.35 |
| 18 | 77282689 | 0.008 | 0.192 | 0 | 0.266 | 0.052 |
| 19 | 13816761 | 0.002 | 0.013 | 0 | 0.304 | 0.022 |
| 20 | 62907 | 0.016 | 0.059 | 0 | 0.388 | 0.05 |

**Supplementary Figure 1 (SF1):** BOILED-Egg plots showing predicted BBB (yolk) and gastrointestinal (white) permeability based on WLOGP and TPSA. A) Standard, B) I1Hit1, C) I2Hit1, D) I1Hit2/I2Hit2

**Supplementary Figure 2 (SF2):** Principal component analysis of protein-ligand complexes, showing eigenvalues plotted against eigenvectors for A) IDH1 and B) IDH2 complexes.

**Supplementary Figure 3 (SF3):** 2-D projection trajectory plot in PCA analysis for the IDH1 protein complex A) IDH1, B) Standard, C) I1Hit1, D) I1Hit2

**Supplementary Figure 4 (SF4):** 2-D projection trajectory plot in PCA analysis for the IDH2 protein complex A) IDH2, B) Standard, C) I2Hit1, D) I2Hit2
